# Learning From Natural Experiments to Accelerate Demographic Research on Climate-Related Threats to Human Populations

**DOI:** 10.1002/wcc.70031

**Published:** 2025-11-23

**Authors:** Elizabeth Fussell, Kate Burrows, Narayan Sastry

**Affiliations:** 1 Population Studies and Training Center and Institute at Brown for Environment and Society, Providence, Rhode Island, USA; 2 Biological Sciences Division, The University of Chicago, Chicago, Illinois, USA; 3 Institute for Social Research, University of Michigan, Ann Arbor, Michigan, USA

**Keywords:** demography, disasters, fertility, migration, mortality

## Abstract

Climate change has increased the destructive force of natural hazards and the occurrence of disasters that damage housing and infrastructure and threaten the health and wellbeing of human populations. Demographic research on disasters advances understanding of climate impacts on coastal populations exposed to tropical cyclones, storm surge, and flooding. However, rigorous study designs that allow for inferences about causal mechanisms are needed. We review natural experimental study design elements and research findings from four demographic surveys disrupted by large-scale disasters. Two studies focus on the effects of Hurricane Katrina on New Orleans’ population and the other two on the 2004 Indian Ocean and 2011 Tōhoku Tsunami effects in Indonesia and Japan, respectively. Three elements of the studies’ designs support the most novel findings: measures of pre- and post-disaster demographic and health outcomes; multiple post-disaster follow-up data waves; and measures of variability in hazard exposure and impact. Two other design elements, population representation and the existence of an unexposed control group, are less critical. Study results advanced knowledge of the effects of damage-related displacement on mental and physical health and wellbeing, as well as harder-to-observe effects of disaster exposure on mortality and fertility outcomes. Acknowledging that natural experiments are rare, we evaluate opportunities for research on hazard exposures and population outcomes using administrative data, existing panel surveys, and new retrospective surveys. Linkage of these data sources to hazard exposure data can expand geographic and population coverage in this field and accelerate understanding of climate-related impacts on population outcomes.

This article is categorized under:

The Social Status of Climate Change Knowledge > Sociology/Anthropology of Climate Knowledge

Assessing Impacts of Climate Change > Observed Impacts of Climate Change

Perceptions, Behavior, and Communication of Climate Change > Behavior Change and Responses

## 1 | Introduction

Climate change has altered weather patterns and increased sea levels, raising the risk that weather events and coastal storms will cause a disaster, especially in coastal communities (Dura et al. 2021; Hanson et al. 2011). While climate change makes these weather events more frequent and destructive and sea level rise intensifies coastal storms and tsunamis, the best models of the human impacts from future climate-related disasters are past disaster events, which damage the built environment and threaten human lives and health (Banwell et al. 2018; Clarke et al. 2022; Ford et al. 2010; Intergovernmental Panel on Climate Change (IPCC) 2023; McLeman and Hunter 2010; Raimi et al. 2017). Disasters occur when natural hazards, such as hurricanes, floods, wildfires, earthquakes, tsunamis and other destructive events overwhelm the coping capacity of the built environment and social systems, often through direct damage to housing and infrastructure (Maxwell et al. 2023). For example, the Camp Fire in Butte County, California, became a human disaster because it crossed the wildfire urban interface and destroyed far more housing than similarly large wildfires that primarily affected uninhabited areas (McConnell et al. 2024). Generally, there are two key mechanisms linking disasters to human population outcomes: loss of and damage to the housing and resources upon which livelihoods depend and the physical and mental trauma that results in death, injury, and distress (McMichael 2017). While not all highly destructive disasters are attributable to climate change, the lessons learned from human responses to geological as well as weather-related hazards have value for improving scientific understanding and designing effective policies and practices to mitigate the impacts of extreme weather events on human health and well-being.

When a major disaster occurs in an area in which a demographic study is underway, a rare opportunity exists to examine the effects of a disaster on fundamental processes shaping population size and composition, specifically, fertility, mortality, health and migration (Arcaya et al. 2020; Hunter et al. 2015; Kemp et al. 2022; McLeman and Hunter 2010; Piguet 2022). Repurposing of such studies allows researchers to investigate whether and for whom disasters affect these demographic and health outcomes. However, disasters present complex challenges affecting every aspect of research, from sampling and instrumentation to data collection and analysis (Galea et al. 2008; Kessler et al. 2008; Norris 2006). Without addressing these challenges, the study design may be compromised, affecting the external and internal validity of the data and undermining statistical estimates (Carleton and Hsiang 2016; Guo et al. 2014). Because of these challenges, scientists rarely have the opportunity to study disasters’ immediate and long-term impacts and variation in these impacts for subgroups within the population.

For this review paper, we examined findings from four recent longitudinal studies interrupted by an environmental disaster that were uniquely suited to study the demographic and health impacts of such events. These demographic surveys had sufficiently large pre-disaster samples to support statistical analyses, multiple health and well-being measures, varied disaster exposure measures, and repeated post-disaster rounds of data collection. These features allow for analysis of a wide range of demographic outcomes over a long period and answer research questions about the long-term demographic effects of environmental disasters. Two of these studies focus on Hurricane Katrina’s impact on New Orleans (USA) residents and the other two focus on tsunamis that struck coastal communities in Indonesia and Japan. Although these studies are hardly reproducible, their quasi-experimental research design and broad topical scope allow for more rigorous results and novel findings on a wide range of topics. We summarize study findings to answer two questions. First, what new insights were gained from using a natural experiment approach to studying the effects of these disasters? Second, which study design elements were most critical for achieving those insights? We find that the most valuable study design elements are the pre- and post-disaster measures of key outcomes, repeated follow-up panels, and measures of variable hazard exposure. To conclude, we use insights gained from the review to consider opportunities for future demographic research on disasters and climate change that, instead of repurposing surveys, leverage existing panel surveys and administrative data to advance research on demographic responses to environmental disasters. Such approaches will accelerate knowledge production and inform policy designs to mitigate the impacts of climate-related disasters.

Our review is motivated by the need to more effectively design quasi-experiments to study the effects of environmental disasters on human populations. We organize the review as follows. In Section 2, we provide background on research designs used in disaster research and in social and health sciences more broadly. In Section 3, we describe the four natural disaster studies that became possible when a disaster struck in locations where one or more rounds of a demographic study had occurred and provide background on the challenges involved in repurposing survey data in this way. In Section 4, we summarize the findings from the analysis of the four studies that provide innovative findings about the effects of disasters on fertility, mortality, migration, and health—the four main subjects of demographic research. (See sidebar.) In Section 5, we review data sources other than interrupted surveys that can be leveraged to systematically investigate climate change-related disaster effects on populations.

## 2 | Research Design to Estimate Causal Relationships

Disaster research is a specialized social science field that provides both practical knowledge for managing disaster response and mitigating deleterious impacts, as well as theoretical insights into social relations during normal and non-normal times (Blaikie et al. 2014). Disaster research frequently employs a case study approach that involves going into the field soon after a disaster strikes and employing qualitative and quantitative research based on convenience samples, which are selected because they are convenient rather than being randomly selected using scientific methods (Phillips 2014). This design is depicted in Panel A of Table 1 (Campbell and Stanley 1963; Meyer 1995). Much can be learned from this pragmatic approach. For example, qualitative research is useful to characterize disaster effects in general and to develop specific hypotheses about pathways relating disaster effects to outcomes. Importantly, it is often the only approach available given the unpredictable nature of disasters, which limits the ability of researchers to plan studies in advance. However, it falls short of what is needed to obtain causal estimates (internal validity) and to generalize about the affected population (external validity) (Campbell and Stanley 1963; Roe and Just 2009).

A gold standard for obtaining causal estimates in population research is a natural or quasi-experimental design that employs population representative longitudinal data on individuals or households (Campbell and Stanley 1963; De Vocht et al. 2021; Meyer 1995; Shadish et al. 2002). (It is quasi-experimental, rather than experimental, because randomization to treatment and control is rarely possible or ethical.) Panel B of Table 1 characterizes this approach. A quasi-experimental design has four desirable elements. First, it involves pre- and post-disaster measures, ideally with observations at multiple time points before and after the disaster to measure pre- and post-disaster trajectories. This feature allows researchers to separate changes in outcomes that are affected by factors other than the disaster (Galea et al. 2008; Meyer 1995; Norris 2006). Second, it includes a measure of disaster exposure, ideally with both subjective and objective measures of the extent of exposure. Measuring variation in exposure improves on the simple exposed–unexposed dichotomy by allowing for the possibility of a dose–response effect and of examining non-linear effects (Galea et al. 2008). Third, a comparison group of unexposed but observationally similar individuals provides a measure of what might have happened in the absence of the disaster (De Vocht et al. 2021; Meyer 1995). A clear comparison group is rarely available because disasters affect specific geographic areas with unique contexts that shape the disaster. In the absence of a comparison group, the ability to characterize variation in exposure within the disaster-affected population is a useful approach to identifying disaster effects. Finally, a population representative sample allows findings to be generalizable to the affected population and, if the sample is sufficiently large, allows for comparisons among sub-groups in the population (Campbell and Stanley 1963; Roe and Just 2009). Most disaster research lacks one or more of these design elements, limiting our ability to make strong inferences about the effects of a disaster on individuals or households, to generalize about disaster effects, and to discern differences in disaster effects within the population.

Fran Norris (Norris 2006), a leading expert on disasters and mental health, reviewed the methods used in 225 research publications in her field. She found that the most common research design was a cross-sectional post-disaster data collection using a convenience sample with a small number of cases. Disaster effects on mental health were typically larger in these case study designs than in those employing longitudinal methods. But even in the studies that include controls for baseline mental health and have representative samples, other elements identified above (i.e., repeated post-disaster observations, unex-posed control group) may be lacking. The need for more rigorous population-based research on disaster impacts is now more widely appreciated, although still challenging to achieve (Galea et al. 2008; Goldmann and Galea 2014; Kessler et al. 2008).

## 3 | Repurposing Survey Data to Overcome Study Design Challenges

Studies of the human impacts of disasters cannot be planned, and therefore have both strengths and weaknesses, which are summarized in Table 2. Most importantly, they provide new information on the population impacts of disasters and the mechanisms through which these effects occur. However, they often lack one of the four key elements of a quasi-experimental research design, as is the case with our selected studies, which are described in greater detail below. For example, three of the four studies (STAR, KATIVA-NOLA, and JAGES) are representative of known pre-disaster populations because of their original study designs; RISK is not because the community college students self-selected into school and into the study. Although both STAR and JAGES have the potential to include unexposed households living in communities that were not affected by the tsunamis that could be used to construct a control group, they have not done so after investigators concluded that none of the other locations for which they had data were sufficiently comparable to the disaster-affected area (Kawachi, personal communication, September 25, 2023; Thomas and Frankenberg, personal communication, September 25, 2023). Control groups were not an option for either KATIVA-NOLA or RISK because Hurricane Katrina affected everyone in the original sampled population. Instead, the four studies used variation within the exposed population to estimate the effect of disaster exposure. Even though all these studies include pre-disaster measures of household and individual-level social, economic, and demographic characteristics, which are useful for characterizing exposure and vulnerability to disasters, some desirable baseline measures may not exist, such as previous disaster exposures and disaster preparedness. A particular strength of all the studies is post-disaster measures of a wide range of disaster-related outcomes, as well as respondent-reported and objective measures of disaster exposure. In addition, all four of the studies received generous funding from federal agencies in the U.S., including the National Institute of Health and the National Science Foundation, as well as private foundations and universities, which supported large teams of researchers that published and presented study findings in journals and conferences.

### 3.1 | Study of the Tsunami Aftermath and Recovery (STAR)

When the December 2004 Indian Ocean tsunami struck Indonesia, demographers collaborated with Statistics Indonesia to use the 2004 wave of a national annual cross-sectional demographic survey that included the tsunami-affected areas of Aceh and North Sumatra as the baseline survey for the longitudinal Study of the Tsunami Aftermath and Recovery (STAR) (Frankenberg et al. 2025). The scale of destruction was tremendous and widespread: an estimated 169,000 people (5% of the population of Aceh) died and damages totalled an estimated 4.5 billion US dollars. In 2005, the study investigators traced individuals and households in the disaster-affected area to their current locations and conducted interviews using a questionnaire that captured key measures of disaster-related impacts and losses (Frankenberg et al. 2012, 2020). The resulting sample had a response rate of 96% of survivors and consists of 7157 households and 28,372 respondents. There are seven rounds of post disaster data collection (2005, 2006, 2007, 2008, 2009, 2014, and 2019) that allow for the study of long-term effects of the disaster. In the follow up rounds, data was collected for new household members, including children and new spouses. Variability in disaster impact is a key feature of the STAR study with 60% of communities experiencing no tsunami-related deaths and nearly two-thirds of community residents dying in the most devastated communities (Frankenberg et al. 2020). More than 21 papers have been published using the STAR data on a wide range of topics, including fertility, mortality, health, and residential mobility (Frankenberg et al. 2025).

### 3.2 | Katrina Impacts on the Vietnamese American Population of New Orleans (KATIVA-NOLA)

In summer 2005, before Hurricane Katrina made landfall on the Gulf Coast, researchers had recently completed interviews with a representative sample of Vietnamese immigrants residing in New Orleans (Bui et al. 2021; VanLandingham et al. 2017; Zhang and Pendley 2017). The original study was designed as a cross-sectional comparison of this group with non-immigrants in Vietnam and Vietnamese immigrants who had returned to Vietnam and the purpose was to understand the effect of migration experience on health and wellbeing. Instead, the pre-disaster study of Vietnamese Americans living in New Orleans was repurposed and named the Katrina Impacts on the Vietnamese American population of New Orleans (Zhang and Pendley 2017). Hurricane Katrina over-topped and breached the city’s levees, resulting in floodwater inundating the below-sea-level city and damaging about 72% of residential dwellings; residents were displaced for at least 6 weeks but often much longer, while waiting for floodwater to be removed, debris to be cleared, utilities restored, and homes repaired (Fussell 2015). Flood damage was especially extensive in the Vietnamese enclave (VanLandingham et al. 2017). The baseline survey included 125 first-generation Vietnamese Americans aged 20–54 and included a wide range of measures of physical and mental health, socioeconomic status, social ties, acculturation, and past migration histories. About two-thirds of the original sample had returned to the area in October 2006 (14 months after Katrina) (Vu et al. 2009). A comparison of the 46 members of the original sample who did not return and were not re-interviewed shows that they were not observationally different in important ways from those who returned and were re-interviewed. The response rate in the baseline round was 74%, with smaller samples in subsequent waves but no statistically significant differences were found between responders and non-responders (Zhang and Pendley 2017). At least 14 articles and book chapters and 2 dissertations analyzed the KATIVA-NOLA data to investigate health and residential mobility outcomes. The small sample precluded statistical analyses of fertility and mortality outcomes.

### 3.3 | Resilience in Survivors of Katrina (RISK)

A study of community college retention and graduation enrolled 1019 New Orleans community college students a year before Hurricane Katrina, collecting a wide range of measures of physical and mental health, economic and social functioning, and neighborhood characteristics (Waters 2016). The original study design targeted low-income parents, resulting in a sample of predominantly African American women aged 18–34. About half the sample was reinterviewed in 2005, just before Hurricane Katrina, and all participants were reinter-viewed after the disaster in 2005–06, 2010, and 2019, with high reinterview rates in all waves (Waters 2016). As already noted, Hurricane Katrina devastated the city of New Orleans, where most of the study participants lived, with 80% of the sample reporting severe home damage, one-third experiencing the death of a friend or relative, spending a mean of 130 days (SD = 121) in temporary housing and moving a mean of 3.6 (SD = 1.5) times in the first year or so after the disaster (Fussell and Lowe 2014; Paxson et al. 2012). Post-disaster waves of data collection included new measures of disaster exposure, residential trajectories, employment histories, household composition, social support, and other topics that were culled from in-depth qualitative interviews that were conducted with sub samples of the larger study population (Waters 2016). The RISK data have been used in more than 30 papers and book chapters, 8 dissertations, and 4 master’s papers, focusing on a range of topics including physical and mental health and residential mobility. However, the age range of the sample meant that there were too few deaths or births to support analyses of mortality or fertility.

### 3.4 | Japan Gerontological Evaluation Study

The Japan Gerontological Evaluation Study (JAGES), a panel study of the elderly in Japan that began data collection in 2003, has been used to study disaster recovery after the 2011 Tōhoku Tsunami affected one of the study sites, Iwanuma, a city with a population of 44,187 in 2010 (JAGES 2022; Hikichi et al. 2016; Okuzono et al. 2022). JAGES includes 40 municipalities all over Japan, enrolling approximately 300,000 adults aged 65 and older in six rounds of data collection in 2003, 2006, 2010, 2013, 2016, and 2020 although not all municipalities contributed in each round (JAGES 2022; Hikichi et al. 2020; Yazawa, Shiba, Hikichi, et al. 2023; Yazawa et al. 2024). For this review, we focus only on the English-language research papers that evaluated the effects of the Iwanuma disaster. This disaster killed 180 residents and damaged 5542 dwellings. The 2010 post-disaster survey had a response rate of 59% of the respondents to the previous survey, resulting in a sample of 5058 residents. The post-disaster questionnaire includes measures of disaster exposure and related losses and residential histories that provided insight into variability in post-disaster outcomes. With each follow-up wave, respondents are lost to follow-up mainly due to death, but also sickness, residential mobility, or other non-response reasons, lowering the overall response rate (Hikichi et al. 2020; Yazawa, Shiba, Okuzono, et al. 2023). Only 4.7% of the sample relocated outside of the study area after the tsunami. The JAGES data set has been analyzed in at least 45 English-language journal articles, with publications focused on mortality, physical and mental health, and residential mobility. The sample selection criteria precluded research on fertility.

### 3.5 | Considerations for Repurposing Existing Survey Data for Disaster Research

To our knowledge, there is no existing guidance for what to do when a disaster occurs in an area with a recently completed survey data collection effort. We seek to fill this gap by summarizing what we have learned from our personal knowledge of and involvement in collaborations focused on finding and producing data to study disaster impacts on populations. Based in survey research methodology (Groves et al. 2009), we identify three critical considerations for assessing the feasibility of repurposing an existing survey for disaster research, which we summarize here. First, the survey sample must have the effective sample size to be able to test hypotheses, which can be a challenge given the small geographic scale of many disasters. This requires not just an adequate count of observations but also design features that maintain the effectiveness of these observations through their independence from each other. Assessments of these requirements can be undertaken through standard power calculations, which are based on estimated effect sizes and effective sample sizes (Groves et al. 2009). Second, the sample should be representative of a known population or at least be representative of a group that is of theoretical interest, rather than a non-representative convenience sample (Galea et al. 2008; Norris 2006). Third, the survey questionnaire should include baseline variables that are of scientific interest to disaster researchers, such as physical and mental health measures, as well as other aspects of social and economic wellbeing (Goldmann and Galea 2014; Norris 2006; Phillips 2014). Other survey characteristics are also important, such as whether study participants have shared recontact information and agreed to be recontacted, and whether geocodes or address-level information allow survey records to be linked to contextual, environmental, or administrative data to supplement or validate survey measures.

Two examples of surveys that met these criteria and were repurposed to study Hurricane Katrina are discussed briefly here. (These were not included in our review because, although both have a wide range of variables, researchers did not analyze them for demographic and health outcomes as thoroughly as the four selected studies.) First, the Panel Study of Income Dynamics (PSID), a national representative US family panel study that began in 1968 and includes 10,000 families, instituted a special survey supplement in 1997 for 500 sample families who lived in the three-state area affected by Hurricane Katrina. The PSID Katrina Supplement collected information on mental and behavioral health geared towards outcome comparisons within the supplement (Cerdá et al. 2011), which covered a subsample that was not representative of the affected region. However, the value of an omnibus, ongoing panel survey such as PSID for studying the effects of large-scale disasters extends beyond the possibility of adding a supplement to cover a specific disaster. A national panel, such as PSID, can be divided between those affected and not affected by a specific disaster event or a broad class of disasters. Comparisons between these two groups (especially after incorporating reweighting to make them more observationally similar) provide a powerful design for studying disaster effects—especially in the long term and for a wide variety of outcomes (Elliott and Howell 2017; Howell and Elliott 2019).

A much larger federal survey, the Current Population Survey (CPS), run by the Census Bureau for the Bureau of Labor Statistics (BLS), added questions for the first year after Hurricane Katrina to identify the geographic locations and living conditions of the Katrina-affected population (Cahoon et al. 2006). BLS researchers who had access to these restricted data, analyzed the labor market outcomes and residential mobility of the Katrina-affected population in the year after the disaster (Groen and Polivka 2008, 2010). While restricted access protects respondents’ identities, it limits researchers’ access to data and any forthcoming results. An additional effort to protect respondents’ privacy involved combining geographic areas into more and less damaged areas that were not geographically identifiable. This made it impossible to draw conclusions about differences in disaster recovery between specific locations. The large scale of Hurricane Katrina justified the effort to add a special module to the CPS, but such efforts may be logistically and politically unlikely in the case of smaller scale events.

Summing up, if a disaster-affected study sample has an adequate effective sample size, represents a known population, and includes valuable baseline measures, then using it to study disaster impacts is not only feasible but is also valuable for scientific knowledge production on a variety of high-priority topics. However, it also requires tremendous mobilization of resources to repurpose a study, as well as to support analyses of the data.

## 4 | Existing Survey Research on Disasters and Demographic and Health Outcomes

The four focal studies demonstrate the type of scientific knowledge gained from repurposing existing data for disaster effects on demographic and health outcomes research. Here, we focus on disaster effects on fertility, mortality, health, and migration, ordering them from the topics with the least evidence to those with the most. In each section, we contextualize the contributions of the selected studies by summarizing existing theories and evidence on disaster effects on each outcome. We then describe how the studies reviewed here advanced knowledge in this field, emphasizing the elements of the study design that allowed for that advancement.

### 4.1 | Disaster Effects on Fertility

A recent review of the sparse empirical literature on disasters and fertility concludes that there is not sufficient evidence to draw general conclusions (Lee et al. 2023). A larger literature on fertility responses to shocks shows that fertility tends to decrease during war and increase when conditions return to normal, creating well-known baby busts and booms (Agadjanian and Prata 2002; Caldwell 2004; Heuveline and Poch 2007; Lindstrom and Berhanu 1999; Van Bavel and Reher 2013). Similarly, fertility tends to increase after rapid-onset short-duration disaster events, such as an earthquake (Carta et al. 2012; Finlay 2009) or hurricane (Cohan and Cole 2002; Seltzer and Nobles 2017). STAR’s long post-disaster observation period allowed researchers to investigate the effect of the Indian Ocean tsunami on post-disaster fertility (Nobles et al. 2015). The researchers found that mothers who lost one or more children in the disaster were significantly more likely to bear additional children. The authors also drew on the multilevel structure of the STAR cohort and investigated the relationship between community-level mortality and post-tsunami fertility, finding that women without children before the tsunami tended to initiate family-building earlier in communities where tsunami-related mortality rates were higher. The authors interpret this as couples’ desires to rebuild communities after the disaster. Neither KATIVA-NOLA nor JAGES had appropriate samples of reproductive age women that allowed for examination of fertility outcomes, and the RISK sample was not representative of the population of reproductive age women in New Orleans.

### 4.2 | Disaster Effects on Immediate and Long-Term Mortality

Research on disaster exposure and mortality investigates two hypotheses about the short- and long-term effects of disasters (Ho et al. 2017; Vaupel and Yashin 1985). In the short term, disasters cause higher mortality among the frailest members of the population, an effect known as mortality displacement or harvesting. In high mortality events in which displacement occurs, in subsequent years, all else being equal, mortality is expected to decline because the average health of survivors is higher. Over a longer period, injuries to survivors may be observed to affect health and longevity, an effect known as scarring. Scarring is evident among survivors if mental and physical health is compromised, particularly among those exposed to the harshest disaster effects and those most vulnerable to disaster effects. Whether displacement or scarring dominates post-disaster population mortality is a function of mortality levels during the disaster event (Costa 2012), and varies by sex, age at exposure, and age at follow-up (Ho et al. 2017). Furthermore, in high mortality disaster events where displacement is evident there may be a crossover, in which the healthier survivors manifest scarring over time as injuries and stressors accumulate (Page and Brass 2001; Song 2010). The few natural or quasi-experimental studies that allow for testing of these hypotheses have the potential to improve post-disaster health care delivery by identifying pre-disaster health conditions and demographic characteristics that heighten vulnerability to both immediate and long-term disaster effects.

The STAR study provides the strongest evidence on mortality displacement and scarring due to its comprehensive quasi-experimental study design elements, especially the longitudinal coverage of all age groups. As already noted, approximately 5% of the population of Aceh, Indonesia—over 160,000 people—perished in the tsunami, although communities in the STAR sample varied in disaster-related mortality from no deaths in 60% of communities to the death of nearly two-thirds of community residents in the most devastated communities (Frankenberg et al. 2020). Consistent with the displacement hypothesis, survivors’ mortality levels in the subsequent 10 years were reduced in inverse proportion to community-level tsunami mortality. Mortality displacement varied by sex, age, and position within the family household. Men aged 15–44 were more likely to survive than children, older adults, and women of the same age, a finding attributed to men’s physical strength and ability to swim (Frankenberg et al. 2011). Among members of the groups more likely to drown in the tsunami, survival was related to receiving help from a co-resident man (aged 15–44) who also survived, and death was related to having more household members who needed help, such as children and older women. These findings illustrate the importance of family composition in a disaster.

Mortality displacement is also evident in JAGES, which focuses exclusively on older adults (age 65+) and has a shorter follow-up period (Aida et al. 2017). Among this vulnerable group, participants living within 1 km from the coast and those with depressive symptoms experienced higher mortality from the tsunami event. Investigators speculate that people suffering from depression may have been less likely to evacuate in response to the one-hour warning ahead of the tsunami wave. Marginally significant differences by gender, height, and social connectedness were also noted: short stature (a proxy for physical strength) and those living with others had a higher risk of mortality and men were at a higher risk of mortality than women.

The STAR and JAGES studies are uniquely suited for investigating disaster effects on mortality. The STAR sample is large and representative of the population and the Indian Ocean Tsunami was severely lethal, both of which led to many observed deaths in the sample. JAGES’ sample of older adults, along with the deadliness of the 2011 tsunami, also resulted in many deaths. The long follow-up period of STAR allowed for evaluation of hypotheses and also revealed unique insights into the role of family assistance in survival. The smaller sample sizes and younger ages of participants in the KATIVA-NOLA and RISK studies worked against having a sufficient number of deaths to investigate long-term mortality effects.

### 4.3 | Complexities in Disaster Effects on Physical and Mental Health

A tremendous body of research shows that stressful events are harmful to a wide range of mental and physical health outcomes (O’Connor et al. 2021; Schneiderman et al. 2005; Thoits 2010). Unsurprisingly, all four of the disaster studies reviewed here demonstrate that greater exposure to disaster-related stressors, such as traumatic events, loss of family and friends, and property damage, is associated with heightened post-traumatic stress, depression, and poor physical health (Frankenberg et al. 2008, 2011, 2012; Kino et al. 2021; Norris et al. 2009; Rhodes et al. 2010; Rhodes et al. 2010; Norris et al. 2009; Kino et al. 2021). In this review we observe that the pre- and post-disaster measures of physical and mental health are essential for estimating causal effects of disaster exposure on post-disaster health, but the most innovative results come from the improved measures of disaster exposure, the large number of demographic and social mediators and moderators, and the repeated follow-up observations that build trajectories of health outcomes. These allow for novel tests of hypotheses derived from theoretical models of disaster effects on health and demographic outcomes.

#### 4.3.1 | Pre-Disaster Health as a Risk Factor for Post-Disaster Health

Poor pre-disaster health is an established risk factor for worsened post-disaster health, although pre-disaster measures rarely exist or are reported retrospectively and are therefore subject to recall bias (Goldmann and Galea 2014; Norris et al. 2002). When pre-disaster health measures are available their inclusion in statistical models allows for estimates of change in health from before to after the disaster, providing an important statistical control. It also addresses potential correlation between disaster exposure and the health outcome to assess whether pre-disaster health allows people to avoid disaster exposure or impact. Three of these studies include pre-disaster measures of mental and physical health (KATIVA-NOLA, RISK, and JAGES). However, they provide inconsistent findings regarding the effects of pre-disaster health on post-disaster health.

Consistent with the expectation that poor pre-disaster mental and physical health predicts poor post-disaster health, analyses of the RISK study found that those with mental illness, lower self-rated health, a diagnosed medical condition, and high body mass index (BMI), were more likely to have poor post-disaster health (Lowe et al. 2020; Paxson et al. 2012; Raker et al. 2019; Rhodes et al. 2010). Focusing on physical health and including baseline controls for health conditions, Lowe et al. (2014) found that study participants who reported loss of loved ones and psychological distress experienced post-disaster increases in headaches, digestive problems, and back problems. Similarly, Arcaya et al. (2017) showed that those experiencing PTSD, more specifically, intrusive reminders of the disaster, were at heightened risk of headaches or migraines in the post-disaster period, after controlling for headache or migraine reports in the pre-disaster period. With five waves of data from RISK on physical and mental health (Zacher et al. 2021), found that the new physical health symptoms that emerged in the context of the disaster were less likely to persist or become chronic than those that emerged either before or well after the disaster. These studies demonstrate the value of having pre-disaster measures as control variables.

Concerns with endogeneity, specifically that poor pre-disaster health is associated with greater exposure to disaster-related stressors, are addressed in Rhodes et al. (2010). They show that some types of pre-disaster resources, particularly car ownership, higher income, and greater social support, allowed study participants to avoid hurricane stressors and losses; however, mental and physical health were not found to significantly affect exposures. Most importantly, they found that regardless of baseline resources, those experiencing similar levels of disaster exposure had similar adverse health consequences. The authors propose that pre-disaster resources were insufficient to protect this sample of vulnerable low-income African American mothers who were directly exposed to this large and highly destructive disaster (Fussell 2012; Lowe et al. 2020).

In contrast, analyses of the KATIVA-NOLA study did not find effects of pre-disaster mental health on post-Katrina PTSD symptoms. However, flood exposure, property damage, and subjective trauma were all associated with higher levels of Katrina-related PTSD 1 year after the disaster (Norris et al. 2009). Two years later, only property damage and social support mattered for mental health (Vu and Vanlandingham 2012). The unique population of New Orleans’ Vietnamese Americans, with a collective history of past trauma from exposure to the Vietnam War and time spent in transition camps before settling in the U.S., may have produced greater levels of social support and resilience. This is consistent with the low levels of mental illness at baseline, which were only temporarily elevated after Katrina (Vu and Vanlandingham 2012). VanLandingham and colleagues (VanLandingham et al. 2017) point out that the resilience of this tight-knit group with strong social institutions stands in sharp contrast to the African-American experience of Hurricane Katrina.

The JAGES study of older adults in Japan collected a large number of health metrics, including cognitive disability, incidence of stroke, hypertension, diabetes, dyslipidemia, BMI, waist circumference, current drinking or smoking, instrumental activities of daily living (IADL), and dimensions of mental health such as depression (Hikichi et al. 2016; Kino et al. 2021; Shiba et al. 2019; Tsuboya et al. 2017). Hikichi et al. (2016) showed that increased dementia symptomatology was associated with post-disaster onset of depressive symptoms, loss of interactions with neighbors, and housing damage. Using an inductive machine learning approach, Shiba et al. (2022) found that those with pre-existing depressive symptoms and lower socioeconomic backgrounds were more vulnerable to post-disaster mental health problems. Further, this novel approach revealed effect heterogeneity among subgroups in the population, suggesting that baseline characteristics may be more salient for some groups than for others, opening up a new line of investigation that may help to explain the contrasting findings of the RISK and KATIVA-NOLA studies.

#### 4.3.2 | Social Support as a Mediator of Post-Disaster Health

An early review of the literature on disaster effects on mental health finds ample evidence that greater social resources are associated with less psychological distress after a disaster (Norris et al. 2002). However, subsequent research indicates that what seems like an obvious relationship is in fact complicated and dynamic, depending on the timing of support receipt, the type of support, and how the recipient perceives the support (Kaniasty 2020; Norris et al. 2002). The effect of social support on physical health is a distinct question, receiving less attention, because the mechanisms behind this relationship are less apparent. Three of the four studies (KATIVA-NOLA, RISK, and JAGES) include pre-disaster measures of social support and health to support investigation into this dynamic relationship. Notably, they each measure social support differently, and find differences in its effects, with remarkably strong effects on physical health and more attenuated effects on mental health.

Given the RISK study’s original focus on college retention and graduation among high-risk community college students, it includes a rich range of pre-disaster measures for use in the study of post-disaster resilience. The 8-item perceived social support scale asks respondents the degree to which they agree with statements such as “I have a trustworthy person I can turn to if I have problems.” The relationship between perceived social support and post-disaster mental health is most comprehensively examined in Lowe et al. (2020), although previous RISK analyses also investigate this relationship (Chan et al. 2015; Paxson et al. 2012; Raker et al. 2019). Lowe et al. (2020) use data on posttraumatic stress symptoms (PTSS) collected 1, 4 and 12 years after the disaster to show three symptom trajectories: initially moderate and decreasing PTSS (69%), initially high and decreasing PTSS (23%), and initially and stably high PTSS (8%). No consistently low PTSS trajectory was found. Higher levels of perceived social support protect against initially high and decreasing PTSS vs. initially moderate and decreasing PTSS in a univariate analysis. However, after controlling for demographic and economic factors, pre-disaster resources and vulnerabilities (including perceived social support), and hurricane exposures, perceived social support becomes statistically insignificant. The authors interpret this result as indicating that pre-disaster mental illness may mediate the relationship between perceived social support and post-disaster PTSS trajectory through social causation or social selection. This interpretation is consistent with Chan et al. (2015) who argue that pre-disaster deficits in social resources indirectly affect longer-term mental health outcomes by increasing the risk or impact of exposure to disaster-related stressors. For example, Lowe et al. (2009) found that the impact of pet loss on post-disaster psychological distress was significantly greater for participants with low levels of pre-disaster support. Another line of research shows that pre-disaster social support is strongly related to posttraumatic growth (Manove et al. 2021).

The KATIVA-NOLA study included social support measures in all data collection years. It was measured as having a relationship that was helpful when the respondent needed to discuss family or personal issues. In 2006, the Louisville Social Support and Social Embeddedness Scale (LSSS) was administered, consisting of 13 items that measure the extent and closeness of the respondent’s social network and degree of satisfaction with support provided by that network. With only two post-disaster waves of data, Vu and Vanlandingham (2012) found that having a helpful relationship in the pre-disaster year was positively related to mental health before Hurricane Katrina and 2 years after the disaster, but not 1 year after the disaster. The lack of relationship in the post-disaster year may be partially explained by the expansion of social support participants experienced as they collectively recovered their homes and community in the wake of the disaster. Using all waves of data, Bui et al. (2021) estimated the effects of helpful relationships and of social support and found that social support during this critical period was positively associated with physical health in all post-disaster years and that helpful relationships were positively associated with mental health in 2010, even after controlling for pre-disaster controls, demographic characteristics, and hurricane exposures. These findings suggest that the receipt of support during the emergency and in the early recovery period positively impacted health and perhaps played a different role than social support during non-disaster periods.

The JAGES study also has robust measures of pre-disaster social support given its focus on health and disease among older adults. It measures social support broadly as community-level social capital with measures of social cohesion (generalized trust, mutual help, and community attachment), social participation (attending sports clubs, hobby groups, and volunteering), and reciprocity (providing emotional support, receiving emotional support, and receiving instrumental support). Analyses of the JAGES sample show that social support is protective for the post-disaster onset of depression (Sasaki et al. 2019), cognitive decline (Hikichi et al. 2020), and preservation of functional capacity (Gero et al. 2020), net of demographic characteristics, pre-disaster health, and disaster-related losses. Notably, social support operates at both the individual and community levels to protect against cognitive decline and preserve functional capacity.

### 4.4 | Disaster-Related Housing Damage Effects on Residential Mobility and Health

In the past two decades, the literature on the effects of environmental disasters on displacement and migration has expanded tremendously, motivated by interest in climate change impacts on mobility. Reviews and meta-analyses of this literature show that the effects of environmental change on migration are heterogeneous, finding evidence that environmental events may increase, decrease, or have no effect on migration patterns (Beine and Jeusette 2021; Hoffmann et al. 2021; Piguet 2022; Sedová et al. 2021). Methodological challenges may compromise some of these study results. Although case studies still dominate as the primary methodological approach, there is a growing utilization of diverse and more robust methodologies (Piguet 2022). These approaches have the potential to shed light on the ways in which hazards impact the built environment and lead to the displacement of residents. In general, differences in the effect of disasters on displacement and migration are largely due to the nature of the destructive force (e.g., coastal surge, tsunami wave, wind, flood, drought, etc.), the geographic extent and type of damage to the built and natural environment, and the vulnerabilities of the exposed population (e.g., livelihoods and economic contexts, built environment adaptations, exposed population characteristics). All four reviewed studies focus on highly destructive hurricane- or tsunami-related flood events causing extensive damage, but only three were analyzed for post-disaster residential mobility outcomes. (Only a small percentage of the JAGES sample relocated; Hikichi et al. 2021) However, all four examine the relationship between housing damage and health, with residential mobility being a factor in that relationship.

#### 4.4.1 | Disaster-Related Housing Damage and Residential Mobility

The STAR study of the 2004 Indonesian tsunami impact on Aceh and North Sumatra focuses on a devastated region with variability in disaster impacts. Gray et al. (2014) find that community damage strongly predicted post-disaster mobility: overall, 20% of adult respondents relocated in 4 months after the tsunami, with up to two-thirds moving in the most severely damaged communities. Migration from damaged communities was less selective on individual and household characteristics than that from undamaged communities. However, migrants from both damaged and undamaged communities shared some characteristics: more highly educated residents were more likely to move, while members of farm households were less likely to move.

In contrast to the STAR study, both KATIVA-NOLA and RISK involve study samples in which everyone moved due to the mandatory evacuation of New Orleans and the flooding that prevented return for weeks afterwards. Analyses therefore focus primarily on whether and when study respondents returned to New Orleans. In the KATIVA-NOLA sample, nearly all (98.7%) reported having evacuated from New Orleans; the mean displacement duration was 5.5 months. The most important determinants of return migration in the multivariate regression were owning a home (versus renting) and being a blue-collar worker (versus unemployed) in the baseline survey. In the bi-variate analysis, married respondents and those with dependent children were more likely to return. These relationships are not significant in the multivariate regression, which is likely because they were strongly correlated with homeownership.

Like the KATIVA-NOLA study, the RISK respondents were interviewed during the 18 months after Katrina (Paxson et al. 2012). About half (49.6%) had returned by the time of the interview. The strongest factor predicting return was a geo-referenced measure of flood exposure at the pre-Katrina address: only 23.9% of returnees experienced flooding compared to 58.7% of non-returnees. Among those exposed to flooding, respondents with children were significantly less likely to return. Among those without flood exposure, African Americans and church attendants were less likely to return, while homeowners and those who lived in the homes of friends or relatives were more likely to return. Another RISK analysis found that, after controlling for flood exposure and other pre-disaster characteristics, return migration was more likely for homeowners than renters, and more likely for market-rate renters than for those living in subsidized housing (Fussell and Harris 2014). These findings indicate greater self-selection into return migration among those unexposed to flooding compared to those whose homes flooded, although the forces of the housing market strongly influenced respondents’ ability to exercise choice. An analysis of complementary qualitative interviews with RISK participants explores institutional, labor market, and social dimensions of study participants’ locational decisions, which provides rich evidence of how disaster survivors make decisions in a rapidly evolving post-disaster context (Asad 2015).

#### 4.4.2 | Housing Damage and Displacement as Mediators of Post-Disaster Health

In 2009, Uscher-Pines (2009) reviewed the sparse literature on the effects of relocation on health among disaster survivors. The review revealed inconsistent patterns of results that could be due to the small number of studies and missing study design elements. More recent reviews reach similar conclusions, although they represent a wider range of geographies (Jang et al. 2021; C. McMichael 2023; Schwerdtle et al. 2020). Uscher-Pines (2009) proposed a conceptual framework in which all disaster survivors suffer from trauma and losses due to the primary disaster, and post-disaster residential mobility adds to those impacts by disrupting healthcare provision, social networks, living conditions, and introducing new psychological stressors. Subsequent empirical studies generally support the hypothesis that post-disaster mobility leads to worse health outcomes, although some notable exceptions show improved health of displaced populations that move away from chronic local hazards (C. McMichael 2023). The primary research question in this field is to identify the possible effects of disaster-related residential mobility on health and plausible mechanisms behind the effects. At the same time, the primary methodological challenge is the endogeneity of migration decisions, when health status may inform migration decisions and therefore be correlated with health outcomes. The four selected studies identify key pathways in which housing damage and displacement to different types of housing and communities affect long-term health.

Although only a small proportion (4.7%) of the JAGES sample of older (65+) Japanese adults relocated after the tsunami, analyses of residential mobility supported the hypothesis that mobility negatively affects health. Using the geriatric depression scale, JAGES researchers found that home loss and damage—which often led to residential mobility—significantly increased depression (Sasaki et al. 2019; Tsuboya et al. 2017). The study also compared non-movers and movers who relocated to three different post-disaster housing types: public temporary housing, existing, or new housing. Overall, movers into public temporary housing were 50% more likely to report depressive symptoms than non-movers (Sasaki et al. 2019), an effect that disappeared by the five-year follow-up (Hikichi et al. 2021); movers into existing or new housing units did not experience increased depressive symptoms. A separate analysis (Shiba et al. 2019) found that housing type largely mediated the associations between home loss and unhealthy changes in physical health measures. Trailer-style temporary homes lacked kitchen facilities, resulting in survivors eating out more frequently or relying on packaged food and associated weight gain. However, individual relocation to private housing was associated with lower instrumental activities of daily living and a higher risk of cognitive impairment compared to no relocation at both follow-up points. The JAGES findings highlight that while post-disaster mobility is generally harmful to health in this older population, distinctive housing types and group or individual relocation pose specific risks that can be addressed to mitigate those harms.

In the RISK study, in which all study participants relocated for at least a brief period, home damage, along with hurricane trauma and bereavement, is associated with psychological distress and post-traumatic stress symptoms approximately 1 and 4 years after the disaster, but not at 12 years post-disaster (Paxson et al. 2012; Raker et al. 2019). While home damage often led to residential mobility, post-disaster housing trajectories differed (Fussell and Lowe 2014). At the first RISK wave, there were three displacement profiles based on study participants’ geographic location, type of residence, number of post-disaster moves, and days spent in temporary housing: returned (24%), relocated (48.5%), and unstably housed (27.6%). Compared to participants who returned, the relocated or unstably housed reported more depressive symptoms and perceived stress. Analyses of both RISK survey and interview data from the first post-disaster year, reveal that the break-up of social support networks was strongly related to psychological distress, but not distance from New Orleans, a proxy for migration (Morris and Deterding 2016). (Home damage was not included in their model.) RISK participants who were distant from network members reported feeling “a lack of belonging” and “inability to fulfill relationship obligations.” In contrast, those who relocated away from social ties were more likely than those who returned to New Orleans to report qualitative improvements in their lives (Bosick 2015).

Like the RISK study, all KATIVA-NOLA study participants were displaced from New Orleans in the first post-Katrina year. Analyses also found a strong relationship between disaster-related property damage and worsened post-Katrina mental health. In the first post-Katrina year, property loss and subjective trauma exposure predicted Katrina-related PTSD symptoms (Norris et al. 2009). Notably, the severity of property damage was more impactful for Vietnamese migrants who had spent time in a transition camp during their emigration from Vietnam, suggesting that this group was particularly vulnerable to home loss-related distress. Vu and Vanlandingham (2012) found that housing damage is predictive of poor mental health, but not physical health, at both the first (2006) and second (2007) waves of data collection. It is the only factor predicting poor mental health in the second wave. VanLandingham et al. (2017) found that Vietnamese Americans were exceptionally robust in their recovery because of both structural and cultural resources accumulated through their pre-disaster experiences of community building.

Analyses of the STAR study found that community-level tsunami damage moderates the effect of housing damage on post-disaster mental health (Frankenberg et al. 2008). Specifically, housing damage was not related to post-traumatic stress reactivity (PTSR) in the most heavily damaged or in undamaged communities. It is only in moderately damaged communities that housing damage increases PTSR. However, damage to land, livestock, or equipment was strongly related to PTSR for all three community damage levels, indicating that livelihood threats were more stressful than housing damage. Analysis of multiple waves of data, shows that residents who continued to live in the same community after the tsunami, those whose homes were destroyed and who also received housing assistance had better psychological health, especially when that help arrived early in the recovery process (Laurito et al. 2022).

All four of the selected studies show that disaster-related housing damage is a key stressor with both mental and physical health consequences. Importantly, housing damage led to relocation in only a minority of the study samples, with strong self-selection effects. These selection effects suggest that survivors’ volition over their relocation and housing outcomes tends to mitigate the negative health effects of home damage. In all four studies, housing damage consistently predicted poor mental health, and to a lesser extent physical health. Differences in health outcomes among movers emerged that reflected the importance of housing type among the relocated, the availability and timing of disaster recovery assistance, the community damage level, and the presence of social network members. In short, disaster survivors vary in the personal and material resources available to recover their housing and health, but all of the studies point to ways in which housing characteristics available to movers and social network access can be leveraged to protect against poor health outcomes.

## 5 | New Directions in Research on Disaster Impacts on Population Outcomes

The review in Section 4 showed that three elements provided the most insight about which design elements contributed most to advancing knowledge about the demographic and health effects of disasters. First, longitudinal data on individuals spanning the disaster period (i.e., pre- and post-disaster measures). Second, surveys with a broad range of measures on individual demographic, health, housing, and socioeconomic status expand the number of topics that can be studied and controls that can be included in multivariate models. Third, nuanced measures of disaster exposure allow for comparisons between individuals in their level of exposure and substitute for random assignment to treatment and control groups. In this section, we identify and discuss data sources that have these elements and we assess their strengths and weaknesses. We summarize the quasi-experimental design elements of these new directions in disaster research methods in Table 3.

### 5.1 | Approaches Applying Repurposed Surveys

As we have learned in this review, repurposing surveys interrupted by a disaster for post-disaster data collection is a powerful approach to study the effects of a specific disaster event because it has many of the elements of a quasi-experimental design (Table 3, row 1). Specifically, samples are often representative of the disaster-affected population if that was part of the original study design; they have many pre-disaster measures that may be relevant to disaster research and new questions specific to the disaster and subsequent demographic, social, and health outcomes may be added, especially self-reported disaster exposures, objective disaster exposure measures, and other contextual variables, can be added if observations can be linked at the address level to external data sources. Survey samples allow for variation in exposure, which may be preferable to the construction of exposed and comparison groups. However, there are notable drawbacks to repurposing surveys to study single disaster events. The new post-disaster data collection is costly to implement and may not be feasible in a post-disaster environment (Phillips 2014; Zhang and Pendley 2017). Further, data collection in crisis settings may pose ethical issues as disaster survivors may be particularly vulnerable and require additional human subjects’ protections (Packenham et al. 2017). These challenges may result in low follow-up response rates, especially if the baseline survey was not designed for re-contacting and re-interviewing respondents. Most critically, however, few studies are available for repurposing after a disaster, which limits scope for replication and generalization of results.

### 5.2 | New Post-Disaster Surveys of Pre-Disaster Populations

A rarely used approach to the study of disaster effects is the implementation of a new survey using a pre-disaster sampling frame and a questionnaire with retrospective questions (Table 3, row 2), such as the Displaced New Orleans Residents Pilot Survey (Peterson et al. 2016; Sastry 2009). Distinct from non-random or non-representative samples of post-disaster populations (Smith and McCarty 2009), such a study enhances the potential for population representation and generalization of study results. It also allows researchers to develop customized survey questions focused on a wide range of disaster-related and other outcomes of interest. It may also be designed to include an exposed and a comparison group, or sub-groups with varying disaster exposures. The downside of this approach is that it depends on the availability of a pre-disaster sampling frame and the ability to trace individuals no longer living in the disaster-exposed area (Kessler et al. 2008). It is also costly, requiring effort for tracing and survey administration. Without pre-disaster data, there is also greater potential for recall bias in retrospective questions about pre-disaster measures that are important for understanding change in demographic, social, and health outcomes attributable to disaster exposure (Groves et al. 2009). While the implementation of such a design is challenging, it offers researchers a great deal of control over the design of the study and room for innovation.

### 5.3 | Longitudinal Surveys

A promising approach that does not involve new data collection is the leveraging of existing longitudinal surveys to study disaster effects (Table 3, row 3). This approach encompasses large national surveys that are panels or repeated cross-sections of the population that can be adapted to examine disaster effects. In addition, harmonized international studies support cross-national analyses of disaster effects. Observations in these longitudinal datasets may be linkable to exposure and event datasets (Boyle et al. 2020).^1^ Household panel surveys have multiple observations on the same households and individuals over many years. Some examples of ongoing panel surveys include the U.S. Panel Study of Income Dynamics (PSID) and its sibling studies^2^ and the U.S. Health and Retirement Survey (HRS) and its family of studies around the world.^3^ Repeated cross-sectional surveys include the American Community Survey (ACS) and Demographic and Health Surveys (DHS).^4^ The sampling designs of these surveys can be assessed to determine whether they can be adapted to be representative of a disaster-affected population. Often, these are omnibus surveys with multiple topics relevant to disaster research which can be used as pre- and post-event measures (e.g., Bell et al. 2019; Elliott and Howell 2017; Howell and Elliott 2019; Rydberg et al. 2015). In some cases, there is the possibility of adding new questions, such as retrospective exposure questions or post-disaster mental health scales (e.g., Cerdá et al. 2011; Frankenberg et al. 2008). Another possibility is conducting supplemental studies focusing on disaster-prone areas, such as the planned Puerto Rico Panel Study of Income Dynamics (PR-PSID), which allows for important comparisons of these areas with national populations. Panel surveys have a natural comparison group between survey participants living within and outside a disaster area at the time of an event. The downside of longitudinal surveys is that there may be too few observations connected to a specific localized natural disaster to take an event-based approach; instead, disaster events may need to be aggregated, resulting in some loss of information on the event or context.

A growing body of research uses longitudinal data to study climate variability and disaster effects on a range of demographic and health outcomes. For example, repeated cross-sectional surveys provide representative data for subnational units, which is highly valuable for studying disaster effects with these data, and include household- and individual-level information on fertility, mortality, and health outcomes, and retrospective reports on migration (e.g., Davenport et al. 2020; Eissler et al. 2019; Mueller et al. 2020; Sastry and Gregory 2013; Thiede et al. 2016, 2022). Studies of aggregated disasters of a similar type provide more generalizable results by measuring the variability in the power of the phenomenon of interest. For example, panel surveys have been used to study the effects of exposure to repeated disasters on migration, an outcome that is best studied by following individuals over time (e.g., Elliott and Howell 2017; Gray and Mueller 2012). Such longitudinal data provide many of the elements of a quasi-experimental design shown in Table 1, Panel B, while also being low-cost and easy to implement for researchers because the data owners—often national statistical offices or government-funded studies— bear the costs of data collection.

### 5.4 | Administrative Data

Like longitudinal studies, administrative data on individuals and households provides another low-cost source of data to study disaster effects on population outcomes. There is a wide range of administrative data sources; here, we focus on three (government data, commercial data, and social media data), which are particularly relevant for disaster research because they contain information on at least one outcome of interest (Table 3, rows 4–6).

#### 5.4.1 | Administrative Data From Government Agencies

In the United States, government data can be obtained from agencies, such as the Census Bureau or the Centers for Medicare and Medicaid Services (CMS), that collect data on outcomes of interest such as health and residential mobility (Committee on Future Information Architectures and Council 2011; Nagaraj et al. 2025). A major advantage of these administrative records is that they are available at all levels of geography—national, state, county, and sometimes sub-county levels—and are arguably representative of a known population. Individual records must have a geolocated address or other geographic information at regular time intervals to be associated with event exposure data. This type of data has a limited number of demographic and health measures and is mainly used to study disaster exposure, residential mobility, mortality, and hospitalizations (e.g., Deryugina and Molitor 2020; Dosa et al. 2010). A major advantage of this approach is that administrative data are systematically collected continuously or at regular intervals for geographically or demographically defined populations. These data can be downscaled to a disaster-affected area to define the exposed population, and a comparison group can be constructed from unexposed but observationally similar populations.

#### 5.4.2 | Administrative Data From Commercial Sources

Administrative commercial data is a broad category, including credit data, cell phone call records, and electronic medical records. These datasets come from credit companies (e.g., Equifax), private insurance companies (e.g., Optum) or health information systems (e.g., Epic Systems), and other data vendors. While these data are not representative of geographically or demographically defined populations, their biases are knowable (e.g., the credit-visible population, cell phone subscribers, insured patients) (e.g., Cowie et al. 2017; DeWaard et al. 2020; Dirkzwager et al. 2006; Lu et al. 2016; Tseliou et al. 2016; Yzermans et al. 2005). These resources provide researchers with a detailed but limited set of personal information on demographic and health outcomes and residential locations and often cover wide geographic areas. Their main disadvantage is that they are costly and analysts do not have control over access.

#### 5.4.3 | Administrative Data From Social Media

Finally, social media data are a unique type of administrative or trace data that have been used for disaster research (e.g., Alexander et al. 2019; Cesare et al. 2018). Data from social media companies such as Meta or Twitter (X) have been employed to study mobility and migration (Martín et al. 2020) as well as broad symptoms of mental health and sentiment following disasters (Mendon et al. 2021). These resources share the strengths of government and commercial administrative data, but with less explicit or implicit understanding of population representation and sources of bias (Langlois 2015).

### 5.5 | Summary of Administrative Data

While the adoption of administrative data in disaster research provides many elements of a quasi-experimental design, the limitations of this approach are not trivial. Access may be restricted to data owners or users who pass background checks and agree to follow procedures that protect against the disclosure of personally identifying information. Proprietary data, such as electronic medical records or cell phone records, may be costly or only available at the owners’ discretion. The measures available on administrative records are limited to the information collected by the agency, which is often narrowly focused on individuals’ demographic and residential information and purpose-oriented measures such as medical diagnoses or cell phone location. Administrative data may be more widely available in higher-income countries in which many government agencies and industries collect data through online or computerized data entry systems. In medium- and low-income countries, administrative data may be biased or non-existent due to non-computerized record keeping. Administrative data does not allow for the customizability of a repurposed survey. For example, it is not possible to add subjective or self-reported measures. The strengths and weaknesses of administrative data are becoming clearer as more researchers engage in using this data for studying disaster effects on population (Cesare et al. 2018; Langlois 2015).

## 6 | Conclusion

Researchers look to past disaster events, especially extremely destructive disasters, for guidance on how future climate conditions will influence disaster effects on demographic, social, and health outcomes (Banwell et al. 2018; Clarke et al. 2022; Ford et al. 2010; Intergovernmental Panel on Climate Change (IPCC) 2023; McLeman and Hunter 2010; Raimi et al. 2017). Yet, disaster research often lacks key design elements that support causal inference (Campbell and Stanley 1963; Meyer 1995; Norris 2006; Phillips 2014). Quasi-experimental designs provide the best evidence of causal effects of disaster exposure, but it is challenging to include all design elements necessary to isolate the effect of the disaster and demonstrate short- and long-term outcomes. We reviewed research findings from four demographic surveys in areas affected by a disaster that were subsequently redesigned as natural experiments to answer two related questions. First, what new insights were gained from using a natural experiment approach to studying the effects of these disasters? Second, which study design elements were most critical for achieving those insights? Given that studies such as these are difficult to plan, we also consider alternative approaches to studying disasters to accelerate knowledge production in this field.

We answer the two questions in tandem because the novel insights stem directly from the study design elements. In our professional judgment, the most insightful study findings come from having multiple rounds of data collection that span the disaster, particularly when these extend multiple years beyond the disaster itself. The repeated waves of follow-up data allowed researchers to construct trajectories of physical and mental health (Hikichi et al. 2016; Lowe et al. 2020; Paxson et al. 2012; Rhodes et al. 2010; Shiba et al. 2022; Vu and Vanlandingham 2012; Zacher et al. 2021), residential mobility (Fussell and Lowe 2014; Gray et al. 2014; Paxson et al. 2012; Raker et al. 2019), and to test mortality-related hypotheses of displacement and scarring (Aida et al. 2017; Frankenberg et al. 2011, 2020; Ho et al. 2017). Multiple follow-up interviews over a long period provide insight into the varying duration of disaster impacts on different outcomes (Frankenberg et al. 2020; Raker et al. 2019; Zacher et al. 2021). The longitudinal design element also allowed researchers to assess how pre-disaster health conditions, social support, and housing type interacted with disaster exposure to mitigate or exacerbate post-disaster outcomes (Arcaya et al. 2017; Fussell and Lowe 2014; Hikichi et al. 2020; Lowe et al. 2020; Rhodes et al. 2010; Sasaki et al. 2019; Shiba et al. 2019, 2022; Tsuboya et al. 2017; Vu and Vanlandingham 2012; Zacher et al. 2021). These effect modifiers provide insight into the types of pre-disaster conditions that correspond to mechanisms behind the widely observed associations of income, race, religion, and other demographic categories with variation in post-disaster outcomes. Such insights are vital to targeting vulnerable populations and designing recovery interventions.

We also learned that the identification of a geographically defined control group for an event-focused analysis was often found to be unconvincing and variation in exposure within the disaster-affected geography was preferable (Kawachi, personal communication, September 25, 2023; Thomas & Frankenberg, personal communication, September 25, 2023). Variation in exposure was defined at the community, household, and individual levels, with each being relevant for outcomes observed at the same level. While the measures of disaster exposure are an improvement over a simple measure of the event date, the variety of ways in which disaster exposure is measured in these studies indicates that disaster exposures are multidimensional and specific to each event. For example, the RISK study measured disaster exposure with a count of eight types of experiences during the disaster and the loss of a family member or friend (Lowe et al. 2020), while STAR measured exposure with community level mortality, satellite imagery of tsunami destruction, and interviews with community leaders (Frankenberg et al. 2011). These examples illustrate that there are multiple pathways from hazard exposure to demographic and health impacts, operating at different levels.

Repurposing ongoing surveys for disaster research is not a practical approach to advancing knowledge on the demographic and health effects of disasters. However, elements of disaster research design, especially those that we identified as key in the four reviewed studies, can be replicated using other data sources. Longitudinal surveys and administrative data with individual- or household-level observations are valuable sources of information when they span a disaster event and have spatial identifiers that allow them to be combined with objective and subjective disaster exposure measures (Boyle et al. 2020; Cesare et al. 2018; Committee on Future Information Architectures & Council, 2011; Langlois 2015; Nagaraj et al. 2025). In the case of rare and extremely destructive large-scale events, new data collection (DNORPS and DNORS) or the addition of questions to existing surveys has proven valuable but also required large investments (STAR; RISK; KATIVA-NOLA; JAGES; CPS; PSID). The linkage of disaster exposure measures to ongoing surveys and administrative data provides a practical, low-cost, systematic approach to disaster effects research that will accelerate knowledge production on this topic (Cesare et al. 2018; Connelly et al. 2016; Harron et al. 2017; Jones et al. 2019; Langlois 2015). We reviewed multiple approaches for assembling data, noting the challenges of implementing new data collection versus repur-posing existing data (Table 3). By assembling and assessing these approaches we aim to mobilize more researchers to identify opportunities and design research approaches that will advance knowledge of climate-related disaster impacts on population health and wellbeing. Importantly, these studies will reveal novel insights on the mechanisms producing variation in disaster impacts as well as variability of these impacts on sub-groups in the population.

## Figures and Tables

**TABLE 1 |:** Research design elements in disaster research.

	Pre-disaster population sample	Pre-disaster measures	Disaster exposure measures	Post-disaster measures	
Assignment to exposure	Sample	Time 1		Time 2	Time 3
A. Case studies					
Exposed	×	×	✓	✓	✓
Unexposed or less exposed	×	×	×	×	×
B. Natural experiments					
Exposed	✓	✓	✓	✓	✓
Unexposed or less exposed	✓	✓	✓	✓	✓

*Note:* A “✓” indicates the element is present and an “×” indicates it is missing. *Source:* Authors’ rendition of materials on natural experiments (Campbell and Stanley 1963; Meyer 1995; Phillips 2014; Shadish et al. 2002).

**TABLE 2 |:** Design elements of selected longitudinal studies of disaster-affected populations.

	Pre-disaster population sample	Treatment and comparison groups	Pre-disaster measures	Disaster exposure measures	Post-disaster measures
Study of the Tsunami Aftermath and Recovery (STAR)	✓	×	✓	✓	✓
Katrina Impacts on the Vietnamese American Population of New Orleans (KATIVA-NOLA)	✓	×	✓	✓	✓
Resilience in Survivors of Katrina (RISK)	×	×	✓	✓	✓
Japan Gerontological Evaluation Study (JAGES)	✓	×	✓	✓	✓

*Note:* A “✓” indicates the element is present and an “×” indicates it is missing. *Source:* STAR (Frankenberg et al. 2012, 2020, 2025); KATIVA-NOLA (Bui et al. 2021; VanLandingham et al. 2017; Zhang and Pendley 2017); RISK (Paxson et al. 2012; Rhodes et al. 2010; Waters 2016); JAGES (2022; Hikichi et al. 2016, 2020; Okuzono et al. 2022; Yazawa, Shiba, Okuzono, et al. 2023).

**TABLE 3 |:** Design elements available in survey and administrative data sources for the study of disaster-affected populations.

Data sources	Representative of pre-disaster population	Pre- and post-disaster measures	Disaster exposure measures	Potential for comparison group	Event focused	Implementation
Repurposed surveys	Depends on original survey design	✓	Objective and subjective	✓	✓	New data collection
New post-disaster surveys of pre-disaster populations	✓	Only retrospective reports	Objective and subjective	✓	✓	New data collection
Longitudinal surveys	Depends on original survey design	✓	Objective and subjective	✓	×	No data collection
Administrative data from government agencies	✓	✓	Objective	✓	✓	No data collection
Administrative data from commercial sources	Known biases	✓	Objective	✓	✓	No data collection
Administrative data from social media	Unknown biases	✓	Objective	✓	✓	No data collection

*Note:* Examples of each data source type are found in Section 5. A “✓” indicates the element is present and an “×” indicates it is missing.

## Data Availability

Data sharing is not applicable to this article as no new data were created or analyzed in this study.

## Sidebar Title: Components of Change in the Size, Composition, and Health of a Population

Populations change their size and demographic composition through the combination of fertility, mortality, and in- and out-migration. Fertility and in-migration add members to a geographically defined population, while mortality and out-migration subtract members. The balance of fertility and mortality is called natural growth, while net migration is the balance of in- and out-migration. Fertility and mortality trends tend to change slowly and are related to the overall health of the population. As more infants and children survive to adulthood because of improvements in public health and medicine, the major causes of disease and death shift from communicable to chronic diseases and diseases of old age. Net migration responds to short-term shocks, such as an environmental event or economic or policy change, and its relationship to health is variable and not well studied. The age composition of a population changes along with population trends. For example, as fertility declines, each birth cohort forms a smaller proportion of the population and the median age increases. Similarly, an environmental event that increases the mortality rate among older adults reduces the size of that age group and may lower the median age.

## Footnotes

1 In the U.S. the Spatial Hazards Events and Losses Database for the United States (SHELDUS N.D.) and globally, the Emergency Events Database (EMDAT N.D.) and the United Nations’ DesInventar Sendai provide national disasters inventories of damage and losses from disaster events.

2 These include the U.K.-based Understanding Society (UKHLS), the German Socio-Economic Panel (SOEP), and the Household, Income and Labour Dynamics in Australia (HILDA) Survey, the Swiss Household Panel, the Japan Household Panel Survey, and the Korean Labor & Income Panel Study (KLIPS). Harmonized data from these studies is available through the Cross-National Equivalent File project (https://www.cnefdata.org/).

3 These include the Mexican Health and Aging Study (MHAS), English Longitudinal Study of Aging (ELSA), Survey of Health, Aging, and Retirement in Europe (SHARE; covering 28 countries), China Health and Retirement Longitudinal Study (CHRLS), Longitudinal Aging Study in India (LASI) Japanese Study on Aging and Retirement (JSTAR), and Korean Longitudinal Study of Aging (KLoSA). Harmonized data from these studies is available through the Gateway to Global Aging Data (https://g2aging.org).

4 Harmonized data from ACS, DHS, and other U.S. and international repeated cross-sectional surveys are available through IPUMS (https://www.ipums.org/).

## References

[R1] Agadjanian V, and Prata N. 2002. “War, Peace, and Fertility in Angola.” Demography 39, no. 2: 215–231. 10.1353/dem.2002.0013. 12048949

[R2] Aida J, Hikichi H, Matsuyama Y, 2017. “Risk of Mortality During and After the 2011 Great East Japan Earthquake and Tsunami Among Older Coastal Residents.” Scientific Reports 7, no. 1: 16591. 10.1038/s41598-017-16636-3. 29185489 PMC5707380

[R3] Alexander M, Polimis K, and Zagheni E. 2019. “The Impact of Hurricane Maria on Out-Migration From Puerto Rico: Evidence From Facebook Data.” Population and Development Review 45, no. 3: 617–630.

[R4] Arcaya MC, Lowe SR, Asad AL, Subramanian SV, Waters MC, and Rhodes J. 2017. “Association of Posttraumatic Stress Disorder Symptoms With Migraine and Headache After a Natural Disaster.” Health Psychology: Official Journal of the Division of Health Psychology, American Psychological Association 36, no. 5: 411–418. 10.1037/hea0000433. 27929328 PMC6666314

[R5] Arcaya M, Raker EJ, and Waters MC. 2020. “The Social Consequences of Disasters: Individual and Community Change.” Annual Review of Sociology 46: 671–691. 10.1146/annurev-soc-121919-054827.

[R6] Asad AL 2015. “Contexts of Reception, Post-Disaster Migration, and Socioeconomic Mobility.” Population and Environment 36, no. 3: 279–310. 10.1007/s11111-014-0221-4. 32999520 PMC7523815

[R7] Banwell N, Rutherford S, Mackey B, Street R, and Chu C. 2018. “Commonalities Between Disaster and Climate Change Risks for Health: A Theoretical Framework.” International Journal of Environmental Research and Public Health 15, no. 3: 538. 10.3390/ijerph15030538. 29547592 PMC5877083

[R8] Beine M, and Jeusette L. 2021. “A Meta-Analysis of the Literature on Climate Change and Migration.” Journal of Demographic Economics 87, no. 3: 293–344. 10.1017/dem.2019.22.

[R9] Bell SA, Choi H, Langa KM, and Iwashyna TJ. 2019. “Health Risk Behaviors After Disaster Exposure Among Older Adults.” Prehospital and Disaster Medicine 34, no. 1: 95–97. 10.1017/S1049023X18001231. 30642407 PMC6629521

[R10] Blaikie P, Cannon T, Davis I, and Wisner B. 2014. At Risk: Natural Hazards, People’s Vulnerability and Disasters. 2nd ed. Routledge. 10.4324/9780203714775.

[R11] Bosick SJ 2015. ““Pushed Out on My Own”: The Impact of Hurricane Katrina in the Lives of Low-Income Emerging Adults.” Sociological Perspectives 58, no. 2: 243–263. 10.1177/0731121414561124. 33859452 PMC8046166

[R12] Boyle EH, King ML, Garcia S, Culver C, and Bourdeaux J. 2020. “Contextual Data in IPUMS DHS: Physical and Social Environment Variables Linked to the Demographic and Health Surveys.” Population and Environment 41, no. 4: 529–549. 10.1007/s11111-020-00348-4. 32801411 PMC7428161

[R13] Bui BKH, Anglewicz P, and VanLandingham MJ. 2021. “The Impact of Early Social Support on Subsequent Health Recovery After a Major Disaster: A Longitudinal Analysis.” SSM - Population Health 14: 100779. 10.1016/j.ssmph.2021.100779. 33869723 PMC8040331

[R14] Cahoon LS, Herz DE, Ning RC, 2006. “The Current Population Survey Response to Hurricane Katrina.” Monthly Labor Review. https://www.bls.gov/opub/mlr/2006/article/current-population-survey-response-to-hurricane-katrina.htm.

[R15] Caldwell JC 2004. “Social Upheaval and Fertility Decline.” Journal of Family History 29, no. 4: 382–406. 10.1177/0363199004267744.

[R16] Campbell DT, and Stanley JC. 1963. Experimental and Quasi-Experimental Designs for Research. Rand McNally & Company.

[R17] Carleton TA, and Hsiang SM. 2016. “Social and Economic Impacts of Climate.” Science 353: aad9837. 10.1126/science.aad9837. 27609899

[R18] Carta G, D’Alfonso A, Colagrande I, Catana P, Casacchia M, and Patacchiola F. 2012. “Post-Earthquake Birth-Rate Evaluation Using the Brief Cope.” Journal of Maternal-Fetal & Neonatal Medicine: The Official Journal of the European Association of Perinatal Medicine, the Federation of Asia and Oceania Perinatal Societies, the International Society of Perinatal Obstetricians 25, no. 11: 2411–2414. 10.3109/14767058.2012.697945. 22642571

[R19] Cerdá M, Tracy M, and Galea S. 2011. “A Prospective Population Based Study of Changes in Alcohol Use and Binge Drinking After a Mass Traumatic Event.” Drug and Alcohol Dependence 115, no. 1–2: 1–8. 10.1016/j.drugalcdep.2010.09.011. 20977977 PMC3039709

[R20] Cesare N, Lee H, McCormick T, Spiro E, and Zagheni E. 2018. “Promises and Pitfalls of Using Digital Traces for Demographic Research.” Demography 55, no. 5: 1979–1999. 10.1007/s13524-018-0715-2. 30276667 PMC6292527

[R21] Chan CS, Lowe SR, Weber E, and Rhodes JE. 2015. “The Contribution of Pre- and Postdisaster Social Support to Short-and Long-Term Mental Health After Hurricanes Katrina: A Longitudinal Study of Low-Income Survivors.” Social Science & Medicine (1982) 138: 38–43. 10.1016/j.socscimed.2015.05.037. 26046725

[R22] Clarke B, Otto F, Stuart-Smith R, and Harrington L. 2022. “Extreme Weather Impacts of Climate Change: An Attribution Perspective.” Environmental Research: Climate 1, no. 1: 012001. 10.1088/2752-5295/ac6e7d.

[R23] Cohan CL, and Cole SW. 2002. “Life Course Transitions and Natural Disaster: Marriage, Birth, and Divorce Following Hurricane Hugo.” Journal of Family Psychology: JFP: Journal of the Division of Family Psychology of the American Psychological Association (Division 43) 16, no. 1: 14–25. 10.1037//0893-3200.16.1.14. 11915406

[R24] Committee on Future Information Architectures and Council, 2011. 2011. “Sources and Uses of Data Within the Centers for Medicare and Medicaid Services.” In Strategies and Priorities for Information Technology at the Centers for Medicare and Medicaid Services. National Academies Press (US). https://www.ncbi.nlm.nih.gov/books/NBK189626/. 24600743

[R25] Connelly R, Playford CJ, Gayle V, and Dibben C. 2016. “The Role of Administrative Data in the Big Data Revolution in Social Science Research.” Social Science Research 59: 1–12. 10.1016/j.ssresearch.2016.04.015. 27480367

[R26] Costa DL 2012. “Scarring and Mortality Selection Among Civil War POWs: A Long-Term Mortality, Morbidity and Socioeconomic Follow-Up.” Demography 49, no. 4: 1185–1206. 10.1007/s13524-012-0125-9. 22968939 PMC3496009

[R27] Cowie MR, Blomster JI, Curtis LH, 2017. “Electronic Health Records to Facilitate Clinical Research.” Clinical Research in Cardiology: Official Journal of the German Cardiac Society 106, no. 1: 1–9. 10.1007/s00392-016-1025-6. PMC5226988 27557678

[R28] Davenport F, Dorélien A, and Grace K. 2020. “Investigating the Linkages Between Pregnancy Outcomes and Climate in Sub-Saharan Africa.” Population and Environment 41, no. 4: 397–421. 10.1007/s11111-020-00342-w. 39391542 PMC11465627

[R29] De Vocht F, Katikireddi SV, McQuire C, Tilling K, Hickman M, and Craig P. 2021. “Conceptualising Natural and Quasi Experiments in Public Health.” BMC Medical Research Methodology 21, no. 1: 32. 10.1186/s12874-021-01224-x. 33573595 PMC7879679

[R30] Deryugina T, and Molitor D. 2020. “Does When You Die Depend on Where You Live? Evidence From Hurricane Katrina.” American Economic Review 110, no. 11: 3033–3602. 10.1257/aer.20181026. PMC8340931 34366435

[R31] DeWaard J, Johnson JE, and Whitaker SD. 2020. “Out-Migration From and Return Migration to Puerto Rico After Hurricane Maria: Evidence From the Consumer Credit Panel.” Population and Environment 42, no. 1: 28–42.

[R32] Dirkzwager AJE, Grievink L, van der Velden PG, and Yzermans CJ. 2006. “Risk Factors for Psychological and Physical Health Problems After a Man-Made Disaster. Prospective Study.” British Journal of Psychiatry: the Journal of Mental Science 189: 144–149. 10.1192/bjp.bp.105.017855. 16880484

[R33] Dosa D, Feng Z, Hyer K, Brown LM, Thomas K, and Mor V. 2010. “Effects of Hurricane Katrina on Nursing Facility Resident Mortality, Hospitalization, and Functional Decline.” Disaster Medicine and Public Health Preparedness 4, no. Suppl 1: S28–S32. 10.1001/dmp.2010.11. 23105032 PMC3773950

[R34] Dura T, Garner AJ, Weiss R, 2021. “Changing Impacts of Alaska-Aleutian Subduction Zone Tsunamis in California Under Future Sea-Level Rise.” Nature Communications 12, no. 1: 7119. 10.1038/s41467-021-27445-8. PMC8655008 34880254

[R35] Eissler S, Thiede B, and Strube J. 2019. “Climatic Variability and Changing Reproductive Goals in Sub-Saharan Africa.” Global Environmental Change 57: 101912. 10.1016/j.gloenvcha.2019.03.011. 32818011 PMC7430718

[R36] Elliott JR, and Howell J. 2017. “Beyond Disasters: A Longitudinal Analysis of Natural Hazards’ Unequal Impacts on Residential Instability.” Social Forces 95, no. 3: 1181–1207. 10.1093/sf/sow086.

[R37] Finlay J 2009. “Fertility Response to Natural Disasters: The Case of Three High Mortality Earthquakes (SSRN Scholarly Paper 1372960).” Social Science Research Network. https://papers.ssrn.com/abstract=1372960.

[R38] Ford JD, Keskitalo ECH, Smith T, 2010. “Case Study and Analogue Methodologies in Climate Change Vulnerability Research.” WIREs Climate Change 1, no. 3: 374–392. 10.1002/wcc.48.

[R39] Frankenberg E, Friedman J, Gillespie T, 2008. “Mental Health in Sumatra After the Tsunami.” American Journal of Public Health 98, no. 9: 1671–1677. 10.2105/AJPH.2007.120915. 18633091 PMC2509591

[R40] Frankenberg E, Gillespie T, Preston S, Sikoki B, and Thomas D. 2011. “Mortality, the Family, and the Indian Ocean Tsunami.” Economic Journal (London, England) 121, no. 554: F162–F182. 10.1111/j.1468-0297.2011.02446.x. 25866413 PMC4389648

[R41] Frankenberg E, Nobles J, and Sumantri C. 2012. “Community Destruction and Traumatic Stress in Post-Tsunami Indonesia.” Journal of Health and Social Behavior 53, no. 4: 498–514. 10.1177/0022146512456207. 22940603 PMC3841969

[R42] Frankenberg E, Sumantri C, and Thomas D. 2020. “Effects of a Natural Disaster on Mortality Risks Over the Longer Term.” Nature Sustainability 3, no. 8: 614–619. 10.1038/s41893-020-0536-3. PMC7935047 33681474

[R43] Frankenberg E, Sumantri C, and Thomas D. 2025. “STAR: Study of the Tsunami Aftermath and Recovery.” stardata.org.

[R44] Fussell E 2012. “Help From Family, Friends, and Strangers During Hurricane Katrina: Finding the Limits of Social Networks.” In Displaced: Life in the Katrina Diaspora. University of Texas Press. https://utpress.utexas.edu/9780292737648/.

[R45] Fussell E 2015. “The Long Term Recovery of New Orleans’ Population After Hurricane Katrina.” American Behavioral Scientist 59, no. 10: 1231–1245. 10.1177/0002764215591181. 26880853 PMC4752119

[R46] Fussell E, and Harris E. 2014. “Homeownership and Housing Displacement After Hurricane Katrina Among Low-Income African-American Mothers in New Orleans.” Social Science Quarterly 95, no. 4: 1086–1100. 10.1111/ssqu.12114. 26392638 PMC4573632

[R47] Fussell E, and Lowe SR. 2014. “The Impact of Housing Displacement on the Mental Health of Low-Income Parents After Hurricane Katrina.” Social Science & Medicine 113: 137–144. 10.1016/j.socscimed.2014.05.025. 24866205 PMC4096953

[R48] Galea S, Maxwell AR, and Norris F. 2008. “Sampling and Design Challenges in Studying the Mental Health Consequences of Disasters.” International Journal of Methods in Psychiatric Research 17, no. Suppl 2: S21–S28. 10.1002/mpr.267. 19035439 PMC6879088

[R49] Gero K, Hikichi H, Aida J, Kondo K, and Kawachi I. 2020. “Associations Between Community Social Capital and Preservation of Functional Capacity in the Aftermath of a Major Disaster.” American Journal of Epidemiology 189, no. 11: 1369–1378. 10.1093/aje/kwaa085. 32406501 PMC7604520

[R50] Goldmann E, and Galea S. 2014. “Mental Health Consequences of Disasters.” Annual Review of Public Health 35: 169–183. 10.1146/annurev-publhealth-032013-182435. 24159920

[R51] Gray C, Frankenberg E, Gillespie T, Sumantri C, and Thomas D. 2014. “Studying Displacement After a Disaster Using Large Scale Survey Methods: Sumatra After the 2004 Tsunami.” Annals of the Association of American Geographers. Association of American Geographers 104, no. 3: 594–612. 10.1080/00045608.2014.892351. 24839300 PMC4019446

[R52] Gray C, and Mueller V. 2012. “Natural Disasters and Population Mobility in Bangladesh.” Proceedings of the National Academy of Sciences of the United States of America 109, no. 16: 6000–6005. 10.1073/pnas.1115944109. 22474361 PMC3341015

[R53] Groen JA, and Polivka AE. 2008. “The Effect of Hurricane Katrina on the Labor Market Outcomes of Evacuees.” American Economic Review 98, no. 2: 43–48. 10.1257/aer.98.2.43.

[R54] Groen JA, and Polivka AE. 2010. “Going Home After Hurricane Katrina: Determinants of Return Migration and Changes in Affected Areas.” Demography 47, no. 4: 821–844. 21308560 10.1007/BF03214587 PMC3000040

[R55] Groves RM, Jr FJF, Couper MP, Lepkowski JM, Singer E, and Tourangeau R. 2009. Survey Methodology. John Wiley & Sons.

[R56] Guo Y, Gasparrini A, Armstrong B, 2014. “Global Variation in the Effects of Ambient Temperature on Mortality: A Systematic Evaluation.” Epidemiology 25, no. 6: 781–789. 10.1097/EDE.0000000000000165. 25166878 PMC4180721

[R57] Hanson S, Nicholls R, Ranger N, 2011. “A Global Ranking of Port Cities With High Exposure to Climate Extremes.” Climatic Change 104, no. 1: 89–111. 10.1007/s10584-010-9977-4.

[R58] Harron K, Dibben C, Boyd J, 2017. “Challenges in Administrative Data Linkage for Research.” Big Data & Society 4, no. 2: 2053951717745678. 10.1177/2053951717745678. PMC6187070 30381794

[R59] Heuveline P, and Poch B. 2007. “The Phoenix Population: Demographic Crisis and Rebound in Cambodia.” Demography 44, no. 2: 405–426. 17583312 10.1353/dem.2007.0012 PMC3922767

[R60] Hikichi H, Aida J, Kondo K, 2016. “Increased Risk of Dementia in the Aftermath of the 2011 Great East Japan Earthquake and Tsunami.” Proceedings of the National Academy of Sciences of the United States of America 113, no. 45: E6911–E6918. 10.1073/pnas.1607793113. 27791093 PMC5111665

[R61] Hikichi H, Aida J, Kondo K, and Kawachi I. 2021. “Six-Year Follow-up Study of Residential Displacement and Health Outcomes Following the 2011 Japan Earthquake and Tsunami.” Proceedings of the National Academy of Sciences 118, no. 2: e2014226118. 10.1073/pnas.2014226118. PMC7812822 33397722

[R62] Hikichi H, Aida J, Matsuyama Y, Tsuboya T, Kondo K, and Kawachi I. 2020. “Community-Level Social Capital and Cognitive Decline After a Natural Disaster: A Natural Experiment From the 2011 Great East Japan Earthquake and Tsunami.” Social Science & Medicine 257: 111981. 10.1016/j.socscimed.2018.09.057. 30293854

[R63] Ho JY, Frankenberg E, Sumantri C, and Thomas D. 2017. “Adult Mortality Five Years After a Natural Disaster.” Population and Development Review 43, no. 3: 467–490. 10.1111/padr.12075. 29731526 PMC5929150

[R64] Hoffmann R, Šedová B, and Vinke K. 2021. “Improving the Evidence Base: A Methodological Review of the Quantitative Climate Migration Literature.” Global Environmental Change 71: 102367. 10.1016/j.gloenvcha.2021.102367.

[R65] Howell J, and Elliott JR. 2019. “Damages Done: The Longitudinal Impacts of Natural Hazards on Wealth Inequality in the United States.” Social Problems 66, no. 3: 448–467. 10.1093/socpro/spy016.

[R66] Hunter LM, Luna JK, and Norton RM. 2015. “Environmental Dimensions of Migration.” Annual Review of Sociology 41, no. 1: 377–397. 10.1146/annurev-soc-073014-112223. PMC5979262 29861536

[R67] Intergovernmental Panel on Climate Change (IPCC). 2023. Climate Change 2022 – Impacts, Adaptation and Vulnerability: Working Group II Contribution to the Sixth Assessment Report of the Intergovernmental Panel on Climate Change. Cambridge University Press. 10.1017/9781009325844.

[R68] JAGES. 2022. “Japan Gerontological Evaluation Study.” https://www.jages.net/About-Jages/.

[R69] Jang S, Ekyalongo Y, and Kim H. 2021. “Systematic Review of Displacement and Health Impact From Natural Disasters in Southeast Asia.” Disaster Medicine and Public Health Preparedness 15, no. 1: 105–114. 10.1017/dmp.2019.125. 31959272

[R70] Jones KH, Heys S, Tingay KS, Jackson P, and Dibben C. 2019. “The Good, the Bad, the Clunky: Improving the Use of Administrative Data for Research.” International Journal of Population Data Science 4, no. 1: 587. 10.23889/ijpds.v4i1.587. PMC7479922 32935024

[R71] Kaniasty K 2020. “Social Support, Interpersonal, and Community Dynamics Following Disasters Caused by Natural Hazards.” Current Opinion in Psychology 32: 105–109. 10.1016/j.copsyc.2019.07.026. 31445427

[R72] Kawachi I Personal Communication, September 25, 2023. “Question About Iwanuma Study of Disaster Resilience [Personal Communication].”

[R73] Kemp L, Xu C, Depledge J, 2022. “Climate Endgame: Exploring Catastrophic Climate Change Scenarios.” Proceedings of the National Academy of Sciences of the United States of America 119, no. 34: e2108146119. 10.1073/pnas.2108146119. 35914185 PMC9407216

[R74] Kessler RC, Keane TM, Ursano RJ, Mokdad A, and Zaslavsky AM. 2008. “Sample and Design Considerations in Post-Disaster Mental Health Needs Assessment Tracking Surveys.” International Journal of Methods in Psychiatric Research 17, no. Suppl 2: S6–S20. 10.1002/mpr.269. 19035440 PMC3081100

[R75] Kino S, Aida J, Kondo K, and Kawachi I. 2021. “Persistent Mental Health Impacts of Disaster. Five-Year Follow-Up After the 2011 Great East Japan Earthquake and Tsunami: Iwanuma Study.” Journal of Psychiatric Research 136: 452–459. 10.1016/j.jpsychires.2020.08.016. 32948310 PMC7443179

[R76] Langlois G 2015. “What Are the Stakes in Doing Critical Research on Social Media Platforms?” Social Media + Society 1, no. 1: 2056305115591178. 10.1177/2056305115591178.

[R77] Laurito MM, Frankenberg E, and Thomas D. 2022. “Effects of Housing Aid on Psychosocial Health After a Disaster.” International Journal of Environmental Research and Public Health 19, no. 12: 7302. 10.3390/ijerph19127302. 35742545 PMC9223474

[R78] Lee DS, Batyra E, Castro A, and Wilde J. 2023. “Human Fertility After a Disaster: A Systematic Literature Review.” Proceedings of the Royal Society B: Biological Sciences 290, no. 1998: 0211. 10.1098/rspb.2023.0211. PMC10170212 37161332

[R79] Lindstrom DP, and Berhanu B. 1999. “The Impact of War, Famine, and Economic Decline on Marital Fertility in Ethiopia.” Demography 36, no. 2: 247–261. 10.2307/2648112. 10332615

[R80] Lowe SR, Raker EJ, Waters MC, and Rhodes JE. 2020. “Predisaster Predictors of Posttraumatic Stress Symptom Trajectories: An Analysis of Low-Income Women in the Aftermath of Hurricane Katrina.” PLoS One 15, no. 10: e0240038. 10.1371/journal.pone.0240038. 33085670 PMC7577433

[R81] Lowe SR, Rhodes JE, Zwiebach L, and Chan CS. 2009. “The Impact of Pet Loss on the Perceived Social Support and Psychological Distress of Hurricane Survivors.” Journal of Traumatic Stress 22, no. 3: 244–247. 10.1002/jts.20403. 19462438 PMC3659171

[R82] Lowe SR, Willis M, and Rhodes JE. 2014. “Health Problems Among Low-Income Parents in the Aftermath of Hurricane Katrina.” Health Psychology: Official Journal of the Division of Health Psychology, American Psychological Association 33, no. 8: 774–782. 10.1037/hea0000016. 24295026 PMC4041843

[R83] Lu X, Wrathall DJ, Sundsøy PR, 2016. “Unveiling Hidden Migration and Mobility Patterns in Climate Stressed Regions: A Longitudinal Study of Six Million Anonymous Mobile Phone Users in Bangladesh.” Global Environmental Change 38: 1–7. 10.1016/j.gloenvcha.2016.02.002.

[R84] Manove EE, Poon CYS, Rhodes JE, and Lowe SR. 2021. “Changes in Psychosocial Resources as Predictors of Posttraumatic Growth: A Longitudinal Study of Low-Income, Female Hurricane Katrina Survivors.” Traumatology 27, no. 4: 346–353. 10.1037/trm0000318. 35356133 PMC8962964

[R85] Martín Y, Cutter SL, Li Z, Emrich CT, and Mitchell JT. 2020. “Using Geotagged Tweets to Track Population Movements to and From Puerto Rico After Hurricane Maria.” Population and Environment 42, no. 1: 4–27. 10.1007/s11111-020-00338-6.

[R86] Maxwell K, Marino EK, Eisenhauer E, 2023. “Social Systems and Justice.” In Fifth National Climate Assessment. U.S. Global Change Research Program. https://nca2023.globalchange.gov/chapter/20/.

[R87] McConnell K, Fussell E, DeWaard J, 2024. “Rare and Highly Destructive Wildfires Drive Human Migration in the U.S.” Nature Communications 15, no. 1: 6631. 10.1038/s41467-024-50630-4. PMC11300458 39103334

[R88] McLeman RA, and Hunter LM. 2010. “Migration in the Context of Vulnerability and Adaptation to Climate Change: Insights From Analogues.” Wiley Interdisciplinary Reviews: Climate Change 1, no. 3: 450–461. 10.1002/wcc.51. 22022342 PMC3183747

[R89] McMichael A 2017. Climate Change and the Health of Nations: Famines, Fevers, and the Fate of Populations. Oxford University Press.

[R90] McMichael C 2023. “Climatic and Environmental Change, Migration, and Health.” Annual Review of Public Health 44: 171–191. 10.1146/annurev-publhealth-071421-045148. 36542773

[R91] Mendon S, Dutta P, Behl A, and Lessmann S. 2021. “A Hybrid Approach of Machine Learning and Lexicons to Sentiment Analysis: Enhanced Insights From Twitter Data of Natural Disasters.” Information Systems Frontiers 23, no. 5: 1145–1168. 10.1007/s10796-021-10107-x.

[R92] Meyer BD 1995. “Natural and Quasi-Experiments in Economics.” Journal of Business & Economic Statistics 13, no. 2: 151–161. 10.1080/07350015.1995.10524589.

[R93] Morris KA, and Deterding NM. 2016. “The Emotional Cost of Distance: Geographic Social Network Dispersion and Post-Traumatic Stress Among Survivors of Hurricane Katrina.” Social Science & Medicine 165: 56–65. 10.1016/j.socscimed.2016.07.034. 27494240 PMC5003656

[R94] Mueller V, Sheriff G, Dou X, and Gray C. 2020. “Temporary Migration and Climate Variation in Eastern Africa.” World Development 126: 104704. 10.1016/j.worlddev.2019.104704. 32317824 PMC7173330

[R95] Nagaraj A, Stipanicic F, and Tranchero M. 2025. “Restricted-Access Data in Economics: Adoption, Diffusion, and Impact of U.S. Census Bureau’s Microdata.” Harvard Data Science Review, no. (Special Issue 6). 10.1162/99608f92.19a0e1c7.

[R96] Nobles J, Frankenberg E, and Thomas D. 2015. “The Effects of Mortality on Fertility: Population Dynamics After a Natural Disaster.” Demography 52, no. 1: 15–38. 10.1007/s13524-014-0362-1. 25585644 PMC4411230

[R97] Norris FH 2006. “Disaster Research Methods: Past Progress and Future Directions.” Journal of Traumatic Stress 19, no. 2: 173–184. 10.1002/jts.20109. 16612819

[R98] Norris FH, Friedman MJ, Watson PJ, Byrne CM, Diaz E, and Kaniasty K. 2002. “60,000 Disaster Victims Speak: Part I. An Empirical Review of the Empirical Literature, 1981–2001.” Psychiatry 65, no. 3: 207–239. 10.1521/psyc.65.3.207.20173. 12405079

[R99] Norris FH, Vanlandingham MJ, and Vu L. 2009. “PTSD in Vietnamese Americans Following Hurricane Katrina: Prevalence, Patterns, and Predictors.” Journal of Traumatic Stress 22, no. 2: 91–101. 10.1002/jts.20389. 19235888 PMC2923021

[R100] O’Connor DB, Thayer JF, and Vedhara K. 2021. “Stress and Health: A Review of Psychobiological Processes.” Annual Review of Psychology 72: 663–688. 10.1146/annurev-psych-062520-122331. 32886587

[R101] Okuzono SS, Shiba K, Kim ES, 2022. “Ikigai and Subsequent Health and Wellbeing Among Japanese Older Adults: Longitudinal Outcome-Wide Analysis.” Lancet Regional Health. Western Pacific 21: 100391. 10.1016/j.lanwpc.2022.100391. 35141667 PMC8814687

[R102] Packenham JP, Rosselli RT, Ramsey SK, 2017. “Conducting Science in Disasters: Recommendations From the NIEHS Working Group for Special IRB Considerations in the Review of Disaster Related Research.” Environmental Health Perspectives 125, no. 9: 094503. 10.1289/EHP2378. 28949918 PMC5915198

[R103] Page WF, and Brass LM. 2001. “Long-Term Heart Disease and Stroke Mortality Among Former American Prisoners of War of World War II and the Korean Conflict: Results of a 50-Year Follow-Up.” Military Medicine 166, no. 9: 803–808. 11569446

[R104] Paxson C, Fussell E, Rhodes J, and Waters M. 2012. “Five Years Later: Recovery From Post Traumatic Stress and Psychological Distress Among Low-Income Mothers Affected by Hurricane Katrina.” Social Science & Medicine 74, no. 2: 150–157. 10.1016/j.socscimed.2011.10.004. 22137245 PMC3286602

[R105] Peterson CE, Sastry N, Rendall MS, Ghosh-Dastidar B, and Gregory J. 2016. The Displaced New Orleans Residents Survey Overview, User’s Guide, and Codebook. RAND Corporation. https://www.rand.org/pubs/tools/TL171.html.

[R106] Phillips BD 2014. Qualitative Disaster Research. Oxford University Press.

[R107] Piguet E 2022. “Linking Climate Change, Environmental Degradation, and Migration: An Update After 10 Years.” WIREs Climate Change 13, no. 1: e746. 10.1002/wcc.746.

[R108] Raimi KT, Stern PC, and Maki A. 2017. “The Promise and Limitations of Using Analogies to Improve Decision-Relevant Understanding of Climate Change.” PLoS One 12, no. 1: e0171130. 10.1371/journal.pone.0171130. 28135337 PMC5279784

[R109] Raker EJ, Lowe SR, Arcaya MC, Johnson ST, Rhodes J, and Waters MC. 2019. “Twelve Years Later: The Long-Term Mental Health Consequences of Hurricane Katrina.” Social Science & Medicine (1982) 242: 112610. 10.1016/j.socscimed.2019.112610. 31677480 PMC8450020

[R110] Rhodes J, Chan C, Paxson C, Rouse CE, Waters M, and Fussell E. 2010. “The Impact of Hurricane Katrina on the Mental and Physical Health of Low-Income Parents in New Orleans.” American Journal of Orthopsychiatry 80, no. 2: 237–247. 10.1111/j.1939-0025.2010.01027.x. 20553517 PMC3276074

[R111] Roe BE, and Just DR. 2009. “Internal and External Validity in Economics Research: Tradeoffs Between Experiments, Field Experiments, Natural Experiments, and Field Data.” American Journal of Agricultural Economics 91, no. 5: 1266–1271.

[R112] Rydberg H, Marrone G, Strömdahl S, and Schreeb J. 2015. “A Promising Tool to Assess Long Term Public Health Effects of Natural Disasters: Combining Routine Health Survey Data and Geographic Information Systems to Assess Stunting After the 2001 Earthquake in Peru.” PLoS One 10, no. 6: e0130889. 10.1371/journal.pone.0130889. 26090999 PMC4475001

[R113] Sasaki Y, Aida J, Tsuji T, 2019. “Pre-Disaster Social Support Is Protective for Onset of Post-Disaster Depression: Prospective Study From the Great East Japan Earthquake & Tsunami.” Scientific Reports 9, no. 1: 19427. 10.1038/s41598-019-55953-7. 31857658 PMC6923367

[R114] Sastry N 2009. “Displaced New Orleans Residents in the Aftermath of Hurricane Katrina: Results From a Pilot Survey.” Organization & Environment 22, no. 4: 395–409. 10.1177/1086026609347183. 20361011 PMC2846814

[R115] Sastry N, and Gregory J. 2013. “The Effect of Hurricane Katrina on the Prevalence of Health Impairments and Disability Among Adults in New Orleans: Differences by Age, Race, and Sex.” Social Science & Medicine 80: 121–129. 10.1016/j.socscimed.2012.12.009. 23321678 PMC3640812

[R116] Schneiderman N, Ironson G, and Siegel SD. 2005. “STRESS AND HEALTH: Psychological, Behavioral, and Biological Determinants.” Annual Review of Clinical Psychology 1: 607–628. 10.1146/annurev.clinpsy.1.102803.144141. PMC2568977 17716101

[R117] Schwerdtle PN, McMichael C, Mank I, Sauerborn R, Danquah I, and Bowen KJ. 2020. “Health and Migration in the Context of a Changing Climate: A Systematic Literature Assessment.” Environmental Research Letters 15, no. 10: 103006. 10.1088/1748-9326/ab9ece.

[R118] Sedová B, Čizmaziová L, and Cook A. 2021. “A Meta-Analysis of Climate Migration Literature [Application/Pdf].” CEPA Discussion Papers 29: 83. 10.25932/PUBLISHUP-49982.

[R119] Seltzer N, and Nobles J. 2017. “Post-Disaster Fertility: Hurricane Katrina and the Changing Racial Composition of New Orleans.” Population and Environment 38: 465–490. 10.1007/s11111-017-0273-3. 29200546 PMC5703431

[R120] Shadish WR, Cook TD, and Campbell DT. 2002. Experimental and Quasi-Experimental Designs for Generalized Causal Inference, 623. Houghton, Mifflin and Company.

[R121] Shiba K, Daoud A, Kino S, Nishi D, Kondo K, and Kawachi I. 2022. “Uncovering Heterogeneous Associations of Disaster-Related Traumatic Experiences With Subsequent Mental Health Problems: A Machine Learning Approach.” Psychiatry and Clinical Neurosciences 76, no. 4: 97–105. 10.1111/pcn.13322. 34936171 PMC9102396

[R122] Shiba K, Hikichi H, Aida J, Kondo K, and Kawachi I. 2019. “Long-Term Associations Between Disaster Experiences and Cardiometabolic Risk: A Natural Experiment From the 2011 Great East Japan Earthquake and Tsunami.” American Journal of Epidemiology 188, no. 6: 1109–1119. 10.1093/aje/kwz065. 30874714 PMC7301774

[R123] Smith SK, and McCarty C. 2009. “Fleeing the Storm(s): An Examination of Evacuation Behavior During Florida’s 2004 Hurricane Season.” Demography 46, no. 1: 127–145. 10.1353/dem.0.0048. 19348112 PMC2831263

[R124] Song S 2010. “Mortality Consequences of the 1959–1961 Great Leap Forward Famine in China: Debilitation, Selection, and Mortality Crossovers.” Social Science & Medicine 71, no. 3: 551–558. 10.1016/j.socscimed.2010.04.034. 20542611

[R125] Thiede B, Gray C, and Mueller V. 2016. “Climate Variability and Inter-Provincial Migration in South America, 1970–2011.” Global Environmental Change: Human and Policy Dimensions 41: 228–240. 10.1016/j.gloenvcha.2016.10.005. 28413264 PMC5389124

[R126] Thiede BC, Randell H, and Gray C. 2022. “The Childhood Origins of Climate-Induced Mobility and Immobility.” Population and Development Review 48, no. 3: 767–793. 10.1111/padr.12482. 36505509 PMC9733713

[R127] Thoits PA 2010. “Stress and Health: Major Findings and Policy Implications.” Journal of Health and Social Behavior 51, no. Suppl: S41–S53. 10.1177/0022146510383499. 20943582

[R128] Thomas D, and Frankenberg E. Personnal Communication, September 25, 2023. “Question About STAR Analyses [Personal Communication].”

[R129] Tseliou F, Maguire A, Donnelly M, and O’Reilly D. 2016. “The Impact of Childhood Residential Mobility on Mental Health Outcomes in Adolescence and Early Adulthood: A Record Linkage Study.” Journal of Epidemiology and Community Health 70, no. 3: 278–285. 10.1136/jech-2015-206123. 26475920

[R130] Tsuboya T, Aida J, Hikichi H, 2017. “Predictors of Decline in IADL Functioning Among Older Survivors Following the Great East Japan Earthquake: A Prospective Study.” Social Science & Medicine 176: 34–41. 10.1016/j.socscimed.2017.01.022. 28122269 PMC5373851

[R131] Uscher-Pines L 2009. “Health Effects of Relocation Following Disaster: A Systematic Review of the Literature.” Disasters 33, no. 1: 1–22. 10.1111/j.1467-7717.2008.01059.x. 18498372

[R132] Van Bavel J, and Reher DS. 2013. “The Baby Boom and Its Causes: What we Know and What We Need to Know.” Population and Development Review 39, no. 2: 257–288. 10.1111/j.1728-4457.2013.00591.x.

[R133] VanLandingham MJ, VanLandingham M, and Bankston C. 2017. Weathering Katrina: Culture and Recovery Among Vietnamese Americans. 1st ed. Russell Sage Foundation.

[R134] Vaupel JW, and Yashin AI. 1985. “Heterogeneity’s Ruses: Some Surprising Effects of Selection on Population Dynamics.” American Statistician 39, no. 3: 176–185. 12267300

[R135] Vu L, and Vanlandingham MJ. 2012. “Physical and Mental Health Consequences of Katrina on Vietnamese Immigrants in New Orleans: A Pre- and Post-Disaster Assessment.” Journal of Immigrant and Minority Health 14, no. 3: 386–394. 10.1007/s10903-011-9504-3. 21789559

[R136] Vu L, VanLandingham MJ, Do M, and Bankston CL. 2009. “Evacuation and Return of Vietnamese New Orleanians Affected by Hurricane Katrina.” Organization & Environment 22, no. 4: 422–436. 10.1177/1086026609347187. 20871780 PMC2943234

[R137] Waters MC 2016. “Life After Hurricane Katrina: The Resilience in Survivors of Katrina (RISK) Project.” Sociological Forum 31, no. S1: 750–769. 10.1111/socf.12271. 32999529 PMC7523803

[R138] Yazawa A, Hikichi H, Shiba K, 2024. “Association of Disaster-Related Damage With Inflammatory Diet Among Older Survivors of the Great East Japan Earthquake and Tsunami.” British Journal of Nutrition 131, no. 9: 1648–1656. 10.1017/S0007114524000217. 38258409 PMC11042994

[R139] Yazawa A, Shiba K, Hikichi H, 2023. “Post-Disaster Mental Health and Dietary Patterns Among Older Survivors of an Earthquake and Tsunami.” Journal of Nutrition, Health and Aging 27, no. 2: 124–133. 10.1007/s12603-023-1887-z. PMC9982700 36806867

[R140] Yazawa A, Shiba K, Okuzono SS, Hikichi H, and Kawachi I. 2023. “Bidirectional Associations Between Post-Traumatic Stress Symptoms and Sleep Quality Among Older Survivors of the 2011 Great East Japan Earthquake and Tsunami.” Sleep 46, no. 6: zsad106. 10.1093/sleep/zsad106. 37029901 PMC10465083

[R141] Yzermans CJ, Donker GA, Kerssens JJ, Dirkzwager AJE, Soeteman RJH, and ten Veen PMH. 2005. “Health Problems of Victims Before and After Disaster: A Longitudinal Study in General Practice.” International Journal of Epidemiology 34, no. 4: 820–826. 10.1093/ije/dyi096. 15860632

[R142] Zacher M, Raker EJ, Arcaya MC, Lowe SR, Rhodes J, and Waters MC. 2021. “Physical Health Symptoms and Hurricane Katrina: Individual Trajectories of Development and Recovery More Than a Decade After the Storm.” American Journal of Public Health 111, no. 1: 127–135. 10.2105/AJPH.2020.305955. 33211584 PMC7750613

[R143] Zhang M, and Pendley SC. 2017. Shifting Methods When Disaster Strikes: The Conversion of a Cross-National Comparative Approach to a Longitudinal Cohort Study of Vietnamese Americans. SAGE Publications Ltd. 10.4135/9781526423979.

